# Multifunctional carboxymethyl cellulose-based hydrogels with zwitterionic and silver nanowire components for wound management

**DOI:** 10.1016/j.isci.2025.114336

**Published:** 2025-12-04

**Authors:** Kit-Leong Cheong, Te Pan, Suresh Veeraperumal, Franck Quero, Gowsika Jaikumar, Timo Kikas, Malairaj Sathuvan, Karsoon Tan, Saiyi Zhong, Udayakumar Veerabagu

**Affiliations:** 1 College of Food Science and Technology, Guangdong Ocean University, Zhanjiang 524088, China; 2 Department of Biology, College of Science, Shantou University, Shantou, Guangdong, China; 3 Laboratorio de Nanocelulosa y Biomateriales, Departamento de Ingeniería Química, Biotecnología y Materiales, Facultad de Ciencias Físicas y Matemáticas, Universidad de Chile, Santiago 8370456, Chile; 4 Department of Chemistry, Pachaiyappa’s College, University of Madras, Chennai 600030, India; 5 Chair of Biosystems Engineering, Institute of Forestry and Engineering, Estonian University of Life Sciences, Kreutzwaldi 56, 51014 Tartu, Estonia; 6 Center for Global Health Research, Saveetha Medical College and Hospital, Saveetha Institute of Medical and Technical Sciences, Kancheepuram, Tamil Nadu, India; 7 Guangxi Key Laboratory of Beibu Gulf Biodiversity Conservation, Beibu Gulf University, Qinzhou, Guangxi, China

**Keywords:** Biomaterials, Biotechnology, Cell biology, Materials in biotechnology

## Abstract

Chronic wounds represent a major global health burden, while conventional dressings often fail to meet the complex requirements of chronic wound management, including infection control, moisture balance, and tissue regeneration. In this study, two multifunctional zwitterionic hydrogels—vinylpyridine carboxybetaine/sulfobetaine/silver nanowire/carboxymethyl cellulose (VCS/Ag/CMC) and acrylamide carboxybetaine/sulfobetaine/silver nanowire/carboxymethyl cellulose (ACS/Ag/CMC) – were synthesized. Structural analyses confirmed the successful incorporation of aromatic or aliphatic zwitterionic components and uniform distribution of silver nanowires within the CMC-based hydrogel networks. Both hydrogels exhibited favorable mechanical strength, high swelling capacity, controlled degradability, and sustained silver release, providing a balanced profile for chronic wound applications. *In vitro* studies demonstrated potent antibacterial and antioxidant activities, while *in vivo* experiments revealed accelerated wound closure, enhanced re-epithelialization, collagen deposition, and neovascularization. Moreover, the hydrogels promoted macrophage polarization toward the pro-healing M2 phenotype. Collectively, these findings highlight the promise of zwitterionic hydrogels as multifunctional platforms for chronic wound management and regenerative medicine.

## Highlights

• Dual-zwitterionic CMC hydrogels with Ag nanowires were synthesized and characterized
• Hydrogels show strong antibacterial and antioxidant activity with low cytotoxicity
• Sustained Ag release, robust mechanics, high swelling; tailored for chronic wounds
• Hydrogels speed closure, boost collagen/angiogenesis, shift M2 macrophages

## Graphical abstract

**Figure:**
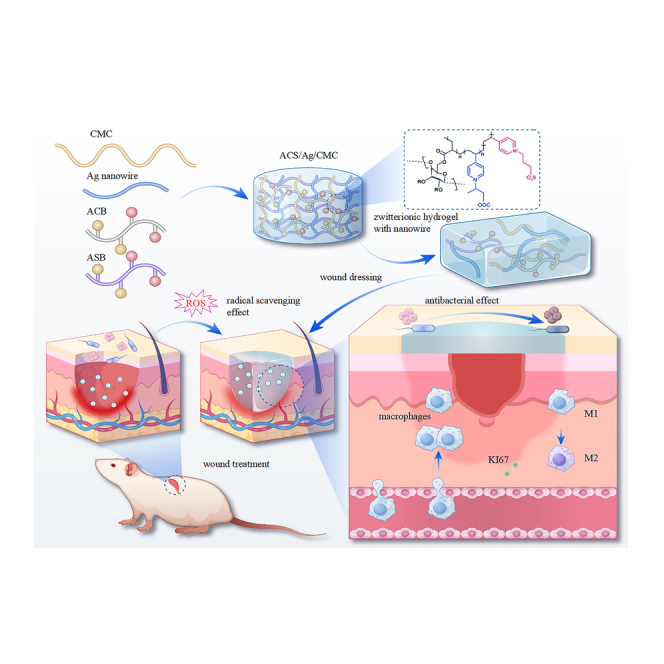

## Introduction

For centuries, wounds have posed a significant concern for patients, placing a heavy burden on healthcare providers and earning the designation silent epidemic.^1^ In developed countries alone, millions of individuals suffer from skin wounds each year, with the frequency increasing in less-developed regions.^2^ Wound healing is a multifaceted biological process critical for restoring skin’s structural integrity after injury. This process involves four distinct, yet interconnected stages: hemostasis, inflammation, proliferation, and remodeling.^3^^,^^4^ Effective healing relies on the precise coordination of various cellular and molecular events, such as clot formation, immune response activation, cell proliferation, extracellular matrix production, and tissue remodeling.^5^^,^^6^ Despite this complex system, numerous factors can interfere with the healing process, resulting in delayed recovery or the formation of chronic wounds. One major hurdle is maintaining immune response balance.^7^ While an adequate inflammatory response is necessary to eliminate pathogens and debris, prolonged or excessive inflammation can hinder healing, leading to scarring or chronic conditions.^8^ Managing moisture levels and ensuring an optimal wound environment are crucial. Insufficient moisture can slow down cell movement, while excessive drainage can lead to tissue breakdown and foster bacterial growth.^9^ Conventional wound care methods, such as gauze dressings, often fall short of meeting these critical needs.^10^ Consequently, there is a rising demand for novel strategies that promote faster healing, minimize complications, and alleviate the strain on both patients and healthcare providers.

Hydrogels are essential in contemporary wound care due to their distinctive features, such as excellent hydration capabilities, biocompatibility, and adaptability in drug delivery.^11^^,^^12^ Made from hydrophilic polymer networks, hydrogels can absorb and retain substantial amounts of water, maintaining a moist environment at the wound site.^13^ This moisture supports cellular processes such as migration and proliferation while preventing the wound from drying out, which is crucial for tissue repair.^14^ Furthermore, hydrogels are biocompatible by nature, reducing the likelihood of immune reactions or irritation when applied.^15^ In addition, the hydrogel can also control the formation of hydrogels with different cross-linking degrees by controlling the amount of cross-linking agent added, which will make the hydrogel have different physical and chemical properties, so that it can be applied to different environments or adhere to various irregular wound surfaces to form a protective layer, thus minimizing mechanical stress and shielding external pollutants.^16^^,^^17^ Hydrogels also function as effective platforms for drug delivery, allowing the controlled release of therapeutic agents such as antibiotics, anti-inflammatory drugs, growth factors, and even nanomaterials.^18^^,^^19^ The addition of silver to hydrogels boosts their effectiveness by facilitating sustained release, localized action, and minimizing systemic toxicity.

Zwitterionic polymers have gained attention as promising candidates in wound care due to their exceptional properties, including resistance to fouling, biocompatibility, and ability to be structurally modified.^20^ These polymers contain both positively and negatively charged groups within the same molecule, which makes them highly effective at preventing fouling by proteins, bacteria, and other bioactive substances.^21^ Their anti-fouling properties stem from the strong hydration layer they form, creating a physical and energetic barrier that inhibits biofilm formation and reduces infection risks.^22^ Zwitterionic polymers are also highly compatible with biological tissues, as their charge neutrality reduces inflammatory responses and enhances tissue interaction.^23^ This makes them particularly beneficial in wound care, where minimizing inflammation and promoting cell function is critical.

**Scheme 1:**
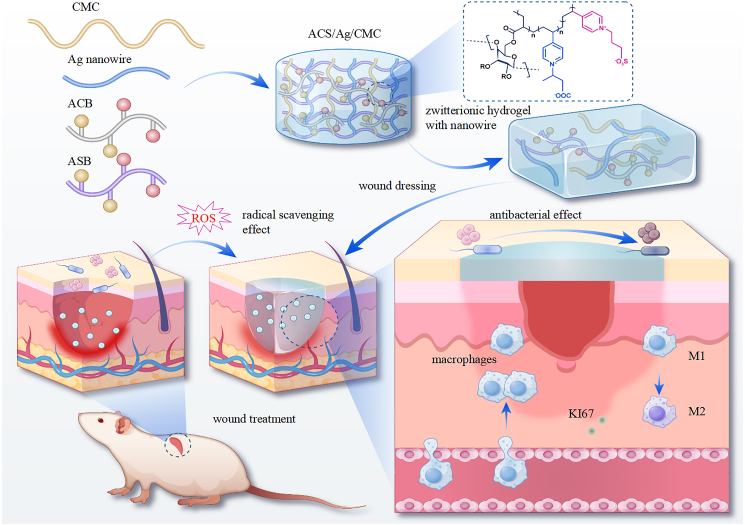
Schematic representation of zwitterionic hydrogels as a multi-functional wound healing dressing The hydrogels exhibit antibacterial, antioxidant, and anti-inflammatory properties, promoting effective wound healing through the synergistic action of these functionalities.

This study aims to develop an innovative hydrogel system designed for enhanced wound management. Carboxymethyl cellulose (CMC) was first modified with acrylic acid to form acrylate-grafted CMC. The modified CMC underwent free radical polymerization with a series of synthesized monomers, including vinylpyridine carboxybetaine/sulfobetaine, acrylamide carboxybetaine/sulfobetaine, and silver nanowires, resulting in the formation of distinct hydrogel formulations. We hypothesize that the development of a polysaccharide-based zwitterionic hydrogel incorporating silver nanowires will provide a multifunctional wound management solution by accelerating wound healing, reducing bacterial infections, and modulating the immune response. Specifically, we propose that the hydrogel system will (1) promote re-epithelialization, collagen deposition, neovascularization, and cell proliferation, (2) exhibit strong antibacterial and antioxidant properties to mitigate infection and oxidative stress, and (3) enhance the immune environment by shifting macrophage polarization from the pro-inflammatory M1 phenotype to the pro-healing M2 phenotype, as illustrated in the Scheme 1. This work demonstrates that silver-functionalized zwitterionic hydrogels offer a synergistic approach to wound management, creating a multifunctional biomaterial capable of accelerating wound healing, reducing complications, and improving clinical outcomes across diverse wound care scenarios.

## Results

### Characterization of vinylpyridine carboxybetaine/sulfobetaine/silver nanowire/carboxymethyl cellulose and acrylamide carboxybetaine/sulfobetaine/silver nanowires/carboxymethyl cellulose zwitterionic hydrogels

#### X-ray diffraction

**Scheme 2:**
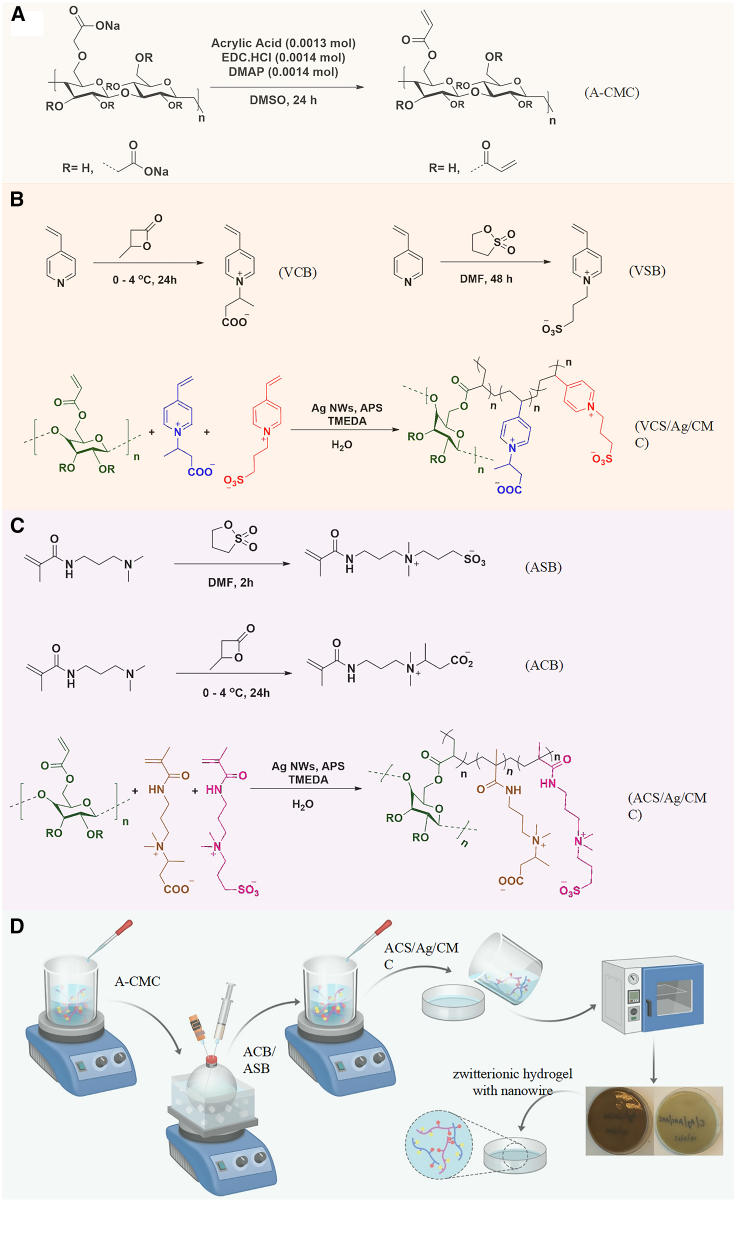
Schematic illustration of the preparation protocol for zwitterionic hydrogels (A) Preparation of acrylate-CMC. (B) Preparation of vinylpyridine carboxybetaine/sulfobetaine/silver nanowire/carboxymethyl cellulose (VCS/Ag/CMC) hydrogel. (C) Preparation of acrylamide carboxybetaine/sulfobetaine/silver nanowires/carboxymethyl cellulose (ACS/Ag/CMC) hydrogel. (D) Synthesis route of the preparation protocol for zwitterionic hydrogels.

**Figure 1:**
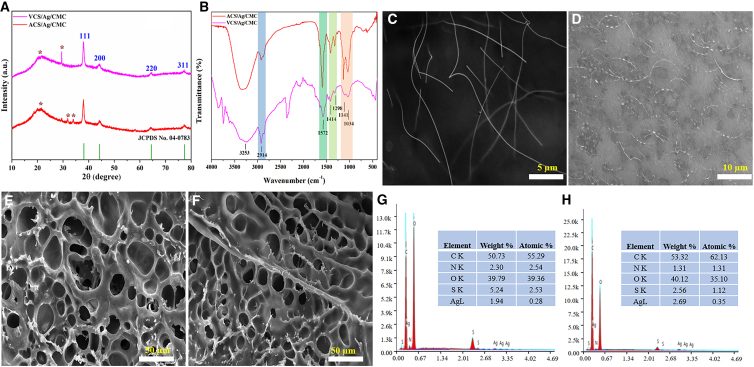
Characterization of VCS/Ag/CMC and ACS/Ag/CMC zwitterionic hydrogels (A) XRD patterns and (B) FTIR spectra. SEM images of oven-dried hydrogels: (C) VCS/Ag/CMC (scale bars = 5 μm) and (D) ACS/Ag/CMC (scale bars = 10 μm). SEM images of freeze-dried hydrogels: (E) VCS/Ag/CMC (scale bars = 50 μm) and (F) ACS/Ag/CMC (scale bars = 50 μm). EDX spectra: (G) VCS/Ag/CMC and (H) ACS/Ag/CMC. See also Figures S1–S11.

XRD analysis was conducted to characterize the crystalline structure of silver-functionalized aromatic and aliphatic zwitterionic hydrogels and to confirm the successful incorporation of silver nanowires into the hydrogel matrices. The XRD patterns of VCS/Ag/CMC and ACS/Ag/CMC hydrogels (Scheme 2) are shown in Figure 1A. Both hydrogels exhibited broad characteristic peaks within the range of 17°–25° (2θ), signifying their predominantly amorphous nature.^24^ This amorphous structure is advantageous for hydrogel applications, as it provides enhanced flexibility and superior swelling properties, both of which are critical for wound-healing performance.

In addition to the amorphous peaks, specific diffraction peaks at 21.1°, 29.4°, 31.9°, and 34.1° were observed, reflecting the fibrillar nature of CMC and its contribution to the structural framework of the hydrogels.^25^ Moreover, distinct peaks at 37.8°, 44.3°, 64.3°, and 77.4° were detected, corresponding to the (111), (200), (220), and (311) crystallographic planes of silver nanowires.^26^ These peaks align well with the face-centered cubic pattern of silver nanoparticles as described by the Joint Committee on Powder Diffraction Standards (JCPDS File No. 04-0783, ASTM). The presence of these silver-specific peaks confirms the successful integration of silver nanowires into the VCS/Ag/CMC and ACS/Ag/CMC hydrogel matrices in their crystalline form. This integration enhances the hydrogels’ functional properties, such as antimicrobial activity and structural stability, while creating a favorable environment for rapid wound healing and tissue regeneration.

#### FT-IR

FTIR spectroscopy was employed to identify the functional groups and chemical interactions within the silver nanowire-functionalized zwitterionic hydrogels (Figure 1B). The absorption peak at 3253 cm^−1^, corresponding to the free hydroxyl (O–H) stretching vibrations in CMC,^27^ indicates the presence of hydroxyl groups, which significantly enhance the hydrophilicity and water retention properties of the hydrogels. These properties are crucial for maintaining a moist environment conducive to wound healing. The absorption band at 2914 cm^−1^, attributed to the asymmetric C–H stretching vibrations of methylene groups in the CMC alkyl chains,^28^ reflects the organic backbone structure, which provides mechanical stability and flexibility to the hydrogel network. Further absorption peaks at 1572 cm^−1^ and 1298–1414 cm^−1^ were assigned to the asymmetric and symmetric stretching vibrations of the carboxylate (COO^−^) groups,^29^ essential for the hydrogel’s zwitterionic nature, charge balance, and ion-exchange properties. These functionalities enable interactions with biological molecules and ions in wound exudates. Additional peaks at 1141 cm^−1^ and 1034 cm^−1^ correspond to the asymmetric and symmetric stretching vibrations of sulfate (SO_3_^−^) groups,^30^ confirming their successful integration into the hydrogel matrix. These sulfate groups improve charge distribution and interactions with biological macromolecules. The FTIR analysis validates the incorporation of key functional groups, including hydroxyl, carboxylate, sulfate, and pyridinium groups, into the hydrogel. These groups collectively enhance the hydrogels’ hydrophilicity, ionic interaction capacity, and chemical stability, ensuring their suitability for advanced wound-healing applications. Their combined effects facilitate moisture retention, biocompatibility, and modulation of ionic interactions within the wound microenvironment.

#### Scanning electron microscopy

SEM was used to examine the surface morphology and microstructure of the hydrogels. Representative images of VCS/Ag/CMC and ACS/Ag/CMC are shown in Figures 1C–1F. To highlight the effect of drying methods, samples were prepared by oven-drying and freeze-drying. In the oven-dried hydrogels, silver nanowires were more clearly visible (Figures 1C and 1D), likely due to the collapse of the polymer network during thermal evaporation, which concentrated and exposed the nanowires on the surface. In contrast, freeze-dried hydrogels displayed a honeycomb-like porous architecture (Figures 1E and 1F), in which the nanowires remained embedded within the polymer walls and were therefore less apparent in surface SEM imaging. The porous morphology enhances oxygen permeability and water retention, both of which are critical for oxygen-dependent tissue regeneration and infection control. Beyond their role in antimicrobial activity, the incorporated silver nanowires also reinforce the hydrogel matrix. The porous, extracellular matrix-like structure supports neovascularization by promoting endothelial cell adhesion and capillary formation,^31^^,^^32^ while also facilitating cell proliferation and migration. In addition, the high surface area of the porous network contributes to superior water-holding capacity.^33^^,^^34^

#### Energy-dispersive X-ray spectroscopy

EDX spectroscopy was performed to verify the elemental composition and distribution of silver nanowires within the aliphatic and aromatic zwitterionic hydrogels. As shown in Figures 1G and 1H and the accompanying elemental table, the spectra provided both qualitative and quantitative evidence of silver incorporation, along with other key elements present in the hydrogel matrices. The analysis revealed carbon (C) at 62.13%, oxygen (O) at 35.00%, sulfur (S) at 1.12%, and silver (Ag) at 0.35%. The high carbon content corresponds to the zwitterionic polymer backbone, which forms the structural framework of the hydrogels and contributes to their mechanical integrity, flexibility, and biocompatibility. The oxygen and sulfur signals reflect the presence of sulfonate and other oxygen-containing functional groups, which improve hydrophilicity and water retention. The detection of silver at 0.35% confirms the successful incorporation of silver nanowires within the hydrogel network, where they are uniformly distributed and strongly bound, supporting their role in antimicrobial activity.^35^ Quantitative verification of silver loading was further obtained by ICP-OES analysis, which showed values of 2.01 wt % for ACS/Ag/CMC and 1.61 wt % for VCS/Ag/CMC hydrogels.

### Mechanical, swelling, degradation, and silver release properties of hydrogels

Mechanical strength, including tensile behavior and elongation at break, is a critical parameter for wound healing applications, as dressings must provide sufficient flexibility and durability to withstand dynamic skin movements. The zwitterionic hydrogels (VCS/Ag/CMC and ACS/Ag/CMC), previously shown to exhibit strong bioactivity in chronic wound healing, were evaluated with and without silver nanowire incorporation. As shown in Figure S11A, the Ag-loaded hydrogels demonstrated significantly improved mechanical performance. The tensile strength of VCS/Ag/CMC and ACS/Ag/CMC reached 0.89 MPa and 0.94 MPa, respectively, compared to 0.71 MPa and 0.72 MPa for their unloaded counterparts (VCS/CMC and ACS/CMC). Similarly, the elongation at break values increased to 52.5% (VCS/Ag/CMC) and 56.3% (ACS/Ag/CMC), exceeding those of the unloaded hydrogels (Figure S11B). The enhanced mechanical performance is attributed to the role of silver nanowires, which act as additional crosslinking sites within the hydrogel matrix while simultaneously forming ionic interactions with hydroxyl, sulfonate, and carboxylate groups of the network. These interactions strengthen the structural framework and improve elasticity, thereby accounting for the higher tensile strength and elongation observed in the Ag-incorporated hydrogels.

The swelling behavior of the synthesized hydrogels is presented in Figure S11C. All formulations exhibited a rapid water uptake during the initial hours of immersion in both distilled water and phosphate buffer at 37°C, followed by a gradual approach to equilibrium. The maximum swelling ratios were nearly stable after 24 h, with only minor changes observed up to 48 h. Notably, the hydrogels showed lower equilibrium swelling in distilled water compared to phosphate buffer, reflecting the influence of ionic strength and buffer interactions on network expansion. Among the formulations, ACS/Ag/CMC demonstrated a slightly higher swelling capacity (550–680%) compared to VCS/Ag/CMC (535–650%). This enhancement can be attributed to the presence of acrylamide-derived ammonium groups, which, in combination with Ag^+^ ions, increase the effective crosslinking density while simultaneously providing additional hydrogen bonding sites. These interactions facilitate greater water retention compared to the vinylpyridinium groups present in VCS/Ag/CMC.

The degradation profiles of the hydrogels were evaluated in phosphate buffer solution over a 28-day period (Figure S11D). A marked weight loss of approximately 70–80% was observed within 21 days for VCB/Ag/CMC, VSB/Ag/CMC, ACB/Ag/CMC, and ASB/Ag/CMC, indicating the relatively rapid degradation of these formulations. In contrast, VCS/Ag/CMC and ACS/Ag/CMC displayed a slower degradation, with 45% and 41% weight loss, respectively, by day 28. Notably, ACS/Ag/CMC exhibited the slowest degradation rate, which can be attributed to the enhanced stability of acrylamide-derived ammonium groups and their stronger ionic interactions with carboxylate and sulfonate functionalities in the hydrogel network. These interactions increase the effective crosslinking stability and reduce chain scission under hydrolytic conditions, thereby retarding degradation compared to the vinylpyridinium-based hydrogel (VCS/Ag/CMC).

The cumulative silver release profiles of the zwitterionic hydrogels were evaluated in phosphate buffer solution (PBS) at 37°C for 24 days (Figure S11E). In the single-component systems (VCB/Ag/CMC, VSB/Ag/CMC, ACB/Ag/CMC, and ASB/Ag/CMC), silver ion release was relatively limited during the first twelve days and then reached a near-constant plateau with minimal further diffusion. In contrast, the dual-zwitterionic formulations, VCS/Ag/CMC and ACS/Ag/CMC, exhibited a continuous and sustained release that persisted throughout the 24-day study. This prolonged retention can be attributed to the synergistic effect of carboxylate, sulfonate, and ammonium functional groups within the hydrogel networks. These groups form stronger electrostatic and coordination interactions with the silver nanowires, thereby stabilizing them within the matrix and delaying ion diffusion. Such controlled and sustained release behavior is expected to maintain long-term antibacterial activity while minimizing burst release, which is beneficial for wound healing applications.

### *In vitro* assessment of cytocompatibility, antibacterial, and antioxidant activities of zwitterionic hydrogels

**Figure 2:**
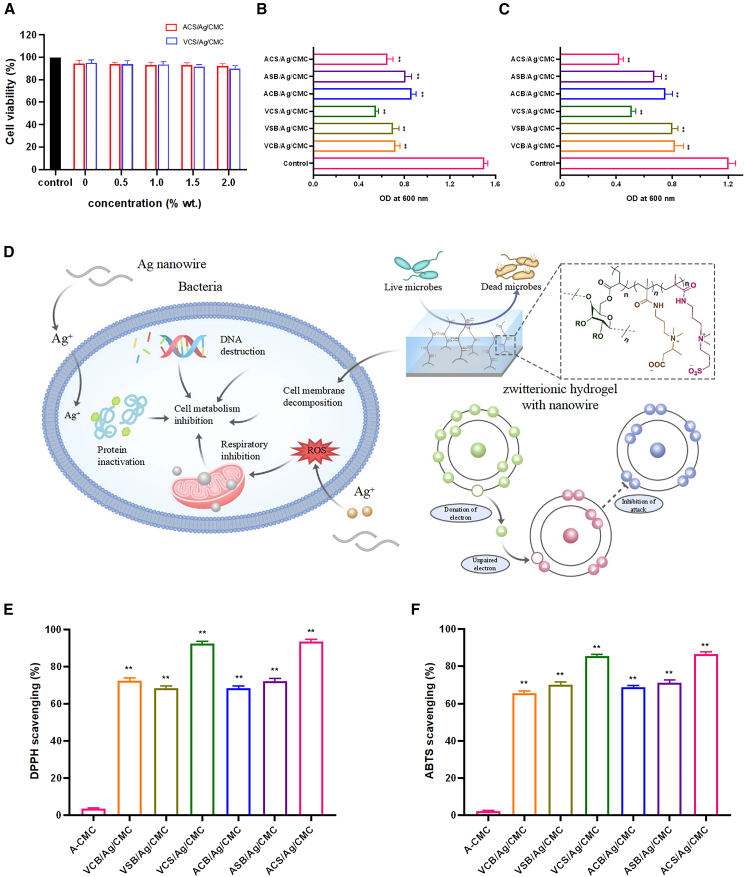
Cytocompatibility, antimicrobial, and antioxidant activity of zwitterionic hydrogels (A) *In vitro* cytotoxicity of zwitterionic hydrogels against L929 cells. Antimicrobial activity of different zwitterionic hydrogels against the Gram-negative bacterium *E. coli* (B) and the Gram-positive bacterium *S. aureus* (C). The potential mechanism of zwitterionic hydrogels in antimicrobial and antioxidant activity (D). Scavenging effect of zwitterionic hydrogels on DPPH radicals (E) and ABTS radicals (F). Data are represented as mean ± SD. There were 3 biological replicates in each group (*n* = 3), and the data points in each biological replicate are the mean of 3 technical replicates. ^∗^*p* < 0.05 and ^∗∗^*p* < 0.01.

The cytotoxicity of VCS/Ag/CMC and ACS/Ag/CMC hydrogels was evaluated in L929 cells. Cells were incubated with each hydrogel formulation for 24 h prior to MTS analysis. The assay, which reflects mitochondrial metabolic activity as an indicator of cell viability and proliferation, showed approximately 90% viability for both hydrogel types (Figure 2A), suggesting minimal cytotoxicity and no abnormal cell growth.

Bacterial infections significantly hinder wound healing, causing delayed tissue regeneration, heightened inflammation, and a risk of chronic wound development.^36^ Antimicrobial hydrogels offer dual advantages: they sustain a moist environment conducive to healing while actively inhibiting bacterial growth and colonization.^37^ The antimicrobial efficacy of the hydrogels was assessed by measuring the OD at 600 nm following bacterial incubation. In the untreated control group, representing *E. coli* growth without hydrogel intervention, a high OD value of 1.5 was recorded, indicating substantial bacterial proliferation. Conversely, the VCS/Ag/CMC and ACS/Ag/CMC hydrogels exhibited marked antibacterial activity. The VCS/Ag/CMC hydrogel reduced the OD to 0.55, while the ACS/Ag/CMC hydrogel yielded an OD of 0.65 (Figure 2B), demonstrating significant inhibition of *E. coli* growth. A similar pattern was observed with *S. aureus*. The VCS/Ag/CMC and ACS/Ag/CMC hydrogels reduced OD values to 0.51 and 0.42 (Figure 2C), respectively, underscoring their potent antibacterial properties. These findings confirm the enhanced antimicrobial performance of silver-functionalized zwitterionic hydrogels compared to the untreated control. The superior antibacterial performance of the hydrogels can be attributed to the controlled release of silver ions (Ag^+^) from their matrix.^38^ These ions disrupt bacterial membranes, impair metabolic activities, and induce oxidative stress within microbial cells.^39^ The slightly enhanced activity observed in aromatic-functionalized hydrogels may result from additional π-π interactions between the aromatic groups and bacterial membranes. These interactions likely facilitate Ag^+^ penetration, thereby intensifying the antimicrobial effect.^40^ The proposed mechanism of this antimicrobial action is illustrated in Figure 2D. Additionally, the zwitterionic characteristics of the hydrogels played a critical role by minimizing biofouling and inhibiting bacterial adhesion on the hydrogel surface, further boosting their antibacterial efficiency.^41^

Reactive oxygen species (ROS) and free radicals are commonly produced during tissue damage and inflammatory responses. Excessive ROS levels can hinder wound healing by causing cellular damage, delaying collagen synthesis, and sustaining chronic inflammation. To evaluate the potential of hydrogels to neutralize ROS in wound environments, their radical-scavenging activity was measured using DPPH and ABTS assays. As illustrated in Figures 2E and 2F, the silver-functionalized zwitterionic hydrogels displayed significantly improved antioxidant activity compared to the control hydrogel (A-CMC), which showed negligible DPPH and ABTS scavenging activities, reflecting minimal intrinsic antioxidant properties. In contrast, all silver-functionalized hydrogels exhibited robust activity, with the zwitterionic variants, VCS/Ag/CMC and ACS/Ag/CMC, demonstrating superior performance. Specifically, VCS/Ag/CMC achieved a DPPH scavenging activity of 92.5%, while ACS/Ag/CMC reached 93.5%. For ABTS radicals, VCS/Ag/CMC and ACS/Ag/CMC showed activities of 86.5% and 85.6%, respectively. These findings highlight the remarkable antioxidant potential of the zwitterionic hydrogels. Their superior radical-scavenging capacity is likely attributed to functional groups capable of donating electrons and the sustained release of silver ions.^42^^,^^43^ Further research has shown that slowly released silver can catalyze hydrogen peroxide (H _2_ O _2_) like catalase.^44^ Therefore, all silver functionalized hydrogels show excellent antioxidant capacity. This enhanced antioxidant performance underscores the hydrogels’ capacity to alleviate oxidative stress, thereby supporting more effective wound healing processes.

### Zwitterionic hydrogels improve wound closure *in vivo* wound

**Figure 3:**
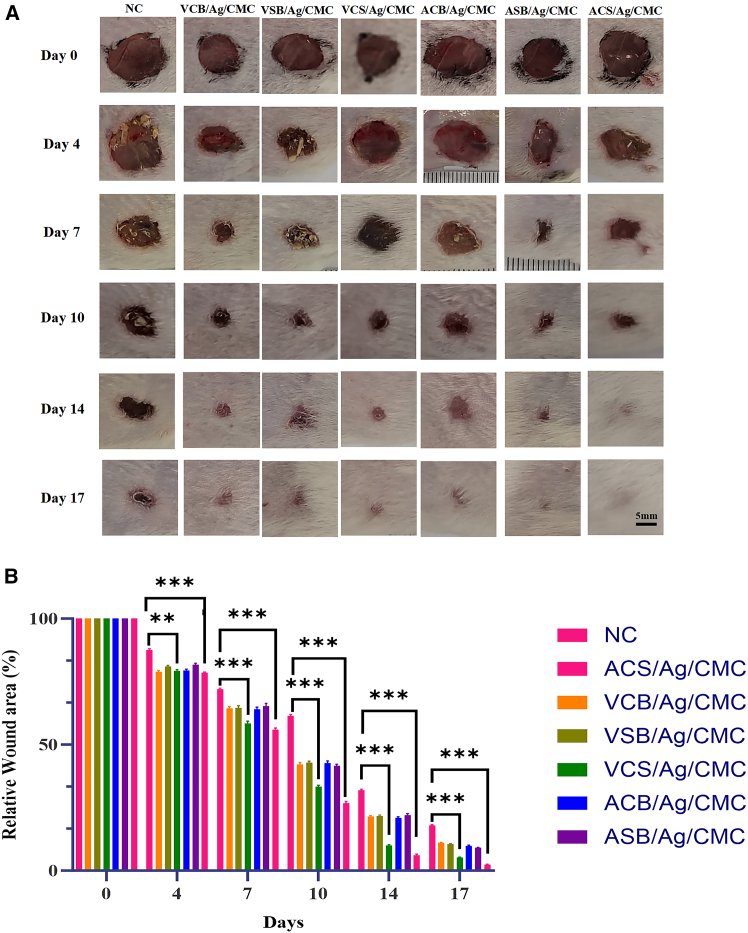
*In vivo* wound healing efficacy of zwitterionic hydrogels (A) Representative photographs of excised wounds treated with zwitterionic hydrogels and saline (control) at days 0, 7, 10, 14, and 17 (scale bars = 5 mm). (B) Quantitative analysis of relative wound closure (%) across all treatment groups, comparing hydrogel-treated wounds to saline-treated controls over the same time points. Data are represented as mean ± SD. Each group at each time point has 8 biological replicates (*n* = 8). ^∗^*p* < 0.05, ^∗∗^*p* < 0.01 and ^∗∗∗^*p* < 0.001.

The *in vivo* wound healing performance of vinylpyridine- and acrylamide-based zwitterionic hydrogels was evaluated using a murine wound model. The rate of wound closure and the quantitative assessment of relative wound bed closure (Figure 3A) were used to determine the wound-healing efficiency of these hydrogels throughout the treatment period.

Compared to the control group, all hydrogel-treated groups (VCB/Ag/CMC, VSB/Ag/CMC, VCS/Ag/CMC, ACB/Ag/CMC, ASB/Ag/CMC, and ACS/Ag/CMC) exhibited enhanced wound regeneration without any visible signs of inflammation or infection around the wound area. Among these, wounds treated with zwitterionic hydrogels (VCS/Ag/CMC and ACS/Ag/CMC) showed significantly faster regeneration compared to other hydrogels. Specifically, after 17 days of treatment, wounds treated with the acrylamide zwitterionic hydrogel had completely healed, leaving minimal to no scarring. In contrast, wounds treated with the vinylpyridine zwitterionic hydrogel still displayed a small residual wound area, despite its superior wound regeneration rate earlier in the treatment period.

As shown in Figure 3B, the restoration area ratio of the wound bed was assessed to evaluate wound healing progress. The final relative wound areas for VCS/Ag/CMC and ACS/Ag/CMC hydrogels were 5.1% and 2.1%, respectively, significantly lower than those of VCB/Ag/CMC (9.8%), VSB/Ag/CMC (10.5%), ACB/Ag/CMC (11.0%), ASB/Ag/CMC (10.09%), and the control group (17.8%). The superior healing performance of the two zwitterionic hydrogels can be attributed to their strong hydration effect via ionic solvation. This arises from their zwitterionic nature, featuring both positively and negatively charged groups on the same molecule. This charge balance enhances their ability to attract and retain water molecules, creating a moist, hydrated environment essential for wound healing.^45^ Sustained hydration promotes cell proliferation, migration, and tissue regeneration while preventing desiccation that can delay healing. In addition, zwitterionic hydrogels are reported to exhibit ionic conductive behavior due to their charged functional groups, which may facilitate electrochemical signaling and cellular communication at the wound site, thereby further supporting bioactivity and therapeutic outcomes.^41^

### Zwitterionic hydrogels improve granulation tissue formation, re-epithelialization, and collagen deposition in the wound bed

**Figure 4:**
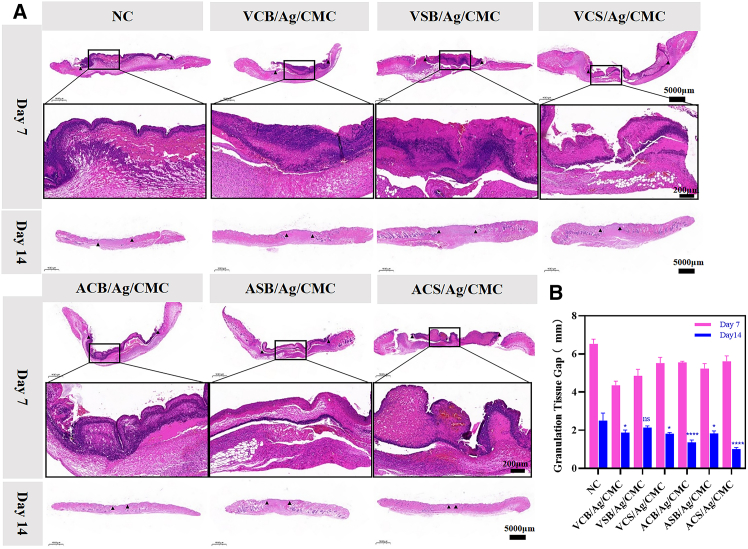
H&E staining of wound sections from zwitterionic hydrogels-treated and control groups at days 7 and 14 post-operation (A) Representative H&E-stained images of skin wounds treated with zwitterionic hydrogels and control at day 7 and day 14, highlighting differences in tissue morphology (day 7 and day 14 images’ scale bars = 5000 μm, and magnified images’ scale bars = 200 μm). (B) Schematic representation of granulation tissue thickness and granulation gap measurements, emphasizing the comprehensive histological analysis conducted to assess the wound healing process. Data are represented as mean ± SD. Each group at each time point has 4 biological replicates (*n* = 4). ^∗^*p* < 0.05, ^∗∗^*p* < 0.01, ^∗∗∗^*p* < 0.001 and ∗∗∗∗*p* < 0.0001.

H & E staining was performed on tissue samples from wounds treated with different hydrogels to assess key histological features of wound healing, including epithelialization, granulation tissue formation, and inflammation. The results revealed distinct tissue responses across treatment groups. As shown in Figure 4A, by day 7, hydrogel-treated wounds displayed nearly complete re-epithelialization, in contrast to the control group, which exhibited delayed epidermal regeneration. By day 14, wounds treated with hydrogels exhibited robust integration of the regenerated dermis with a fully restored epithelial layer, accompanied by the presence of skin appendages. In comparison, the control group still showed unhealed wound regions with a thin granulation layer.

The granulation interval width at days 7 and 14 was significantly reduced in all hydrogel-treated groups except for VSB/Ag/CMC, as shown in Figure 4B. Notably, wounds treated with VCS/Ag/CMC and ACS/Ag/CMC zwitterionic hydrogels demonstrated substantial improvements in epidermal thickness and granulation tissue formation compared to the control. Histological sections of ACS/Ag/CMC-treated wounds at day 14 revealed well-organized, continuous epithelial layers, indicating advanced reepithelialization. The granulation tissue was dense and mature, characterized by abundant fibroblast proliferation, well-formed capillaries, and minimal inflammatory cell infiltration. These results suggest that acrylamide-based zwitterionic hydrogels create an optimal wound environment, likely due to their superior hydrophilicity, biocompatibility, and ability to maintain a moist and bioactive wound microenvironment.^46^

**Figure 5:**
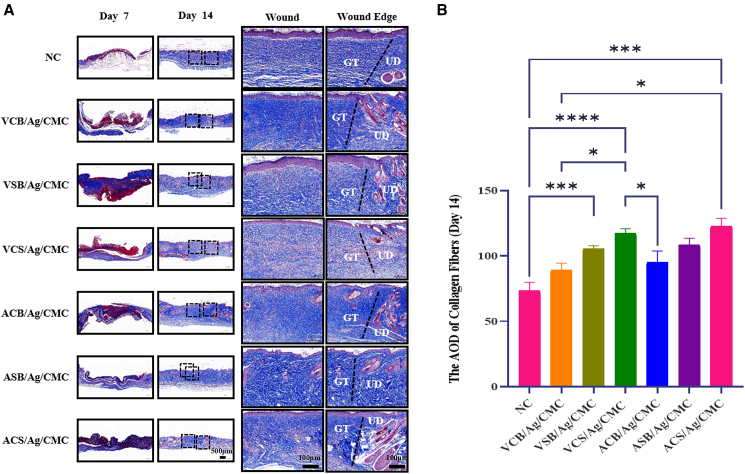
Masson’s trichrome staining of wound sections from zwitterionic hydrogel-treated and control groups on days 7 and 14 post-operation, highlighting collagen deposition and maturation (A) Representative Masson’s trichrome-stained images of skin wounds treated with zwitterionic hydrogels and control at days 7 and 14 (scale bars = 500 μm). Close-up images of the wound center and edge on day 14 are also shown (scale bars = 100 μm). (B) Quantitative analysis of collagen deposition at the wound sites on day 14. Data are represented as mean ± SD. Each group at each time point has 4 biological replicates (*n* = 4). *p* < 0.05, ^∗∗^*p* < 0.01, ^∗∗∗^*p* < 0.001and ∗∗∗∗*p* < 0.0001.

The analysis of collagen deposition and tissue remodeling is critical for evaluating wound healing, as collagen is essential for restoring the structural integrity and functionality of injured tissues.^47^ Masson’s trichrome staining was employed to assess collagen deposition and tissue remodeling in wounds treated with different hydrogels. As shown in Figure 5A, by day 7, hydrogel-treated wounds exhibited a more pronounced azure hue compared to the control group, indicating significantly enhanced collagen deposition. The stained sections revealed abundant blue-stained regions corresponding to mature collagen fibers, which were densely packed and well-aligned, suggesting advanced tissue remodeling and maturation.^48^ Quantitative analysis of collagen deposition on day 14, as illustrated in Figure 5B, further supports these findings. The average optical density values of collagen fibers for the control, VCB/Ag/CMC, VSB/Ag/CMC, VCS/Ag/CMC, ACB/Ag/CMC, ASB/Ag/CMC, and ACS/Ag/CMC groups were 71.8, 89.2, 116.2, 123.9, 94.2, 100.5, and 122.5, respectively. Hydrogels incorporating both carboxybetaine and sulfobetaine groups (VCS/Ag/CMC and ACS/Ag/CMC) outperformed the counterparts formulated with single zwitterionic components, highlighting the synergistic effects of dual zwitterionic functionalities. These groups exhibited higher collagen deposition and better alignment of fibers, highlighting their enhanced capacity for promoting tissue remodeling and maturation. These results validate the efficacy of zwitterionic hydrogels in accelerating wound healing through improved collagen deposition and advanced tissue remodeling.

### Zwitterionic hydrogels promote neovascularization

**Figure 6:**
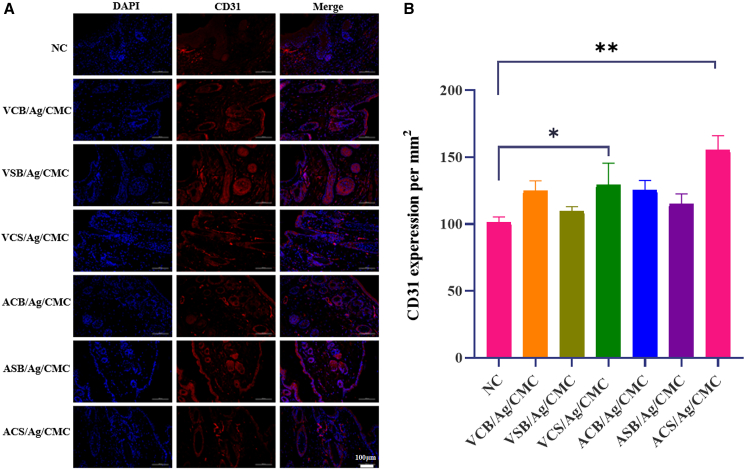
CD31 staining to characterize neovascularization in wound sites of zwitterionic hydrogel-treated and control groups (A) Representative CD31 (red) and DAPI (blue) staining showing newly formed blood vessels on day 14 post-operation (scale bars = 100 μm). (B) Quantitative analysis of neovascularization, measured by vascular area density, at the wound sites on day 14. Data are represented as mean ± SD. Each group has 4 biological replicates (*n* = 4). ^∗^*p* < 0.05 and ∗∗*p* < 0.01.

To further assess angiogenesis during the wound healing process, immunofluorescence staining of CD31 was performed. CD31, also known as platelet endothelial cell adhesion molecule-1, is a specific marker for endothelial cells and is integral to endothelial cell adhesion, migration, and capillary formation.^49^ By evaluating CD31 expression, it is possible to visualize and quantify new blood vessel formation, offering valuable insights into the angiogenic activity stimulated by different treatments or materials.^50^ As illustrated in Figure 6A, the extent of CD31 expression was compared among the experimental groups treated with various hydrogels and the control group. The quantification of the total area occupied by CD31-positive cells, shown in Figure 6B, highlights the concentration of endothelial cells within the wound regions, providing a measure of the angiogenic response elicited by each hydrogel treatment.

The control group exhibited minimal CD31 staining, reflecting baseline angiogenic activity in untreated wounds. Sparse and poorly organized vascular structures observed in this group underscore the crucial role of hydrogel materials in enhancing neovascularization.^51^ In contrast, wounds treated with zwitterionic hydrogels (VCS/Ag/CMC and ACS/Ag/CMC) demonstrated significantly higher CD31 expression relative to wounds treated with hydrogels containing only single zwitterionic functional groups, indicating a superior pro-angiogenic effect of the dual-functionalized systems. This was evidenced by the intense fluorescence signals observed around the wound margins and within the granulation tissue.^52^ The prominent CD31 staining indicates enhanced endothelial cell proliferation and the formation of a dense, well-organized vascular network. The superior angiogenic response observed with the zwitterionic hydrogels can be attributed to their exceptional hydration properties, which maintain a moist wound environment conducive to endothelial cell migration and capillary sprouting.^53^ Among these, ACS/Ag/CMC hydrogels promoted neovascularization more effectively than VCS/Ag/CMC hydrogels, further highlighting their potential in facilitating advanced wound healing.

### Zwitterionic hydrogels promote cell proliferation and polarization of M2/M1 macrophages

**Figure 7:**
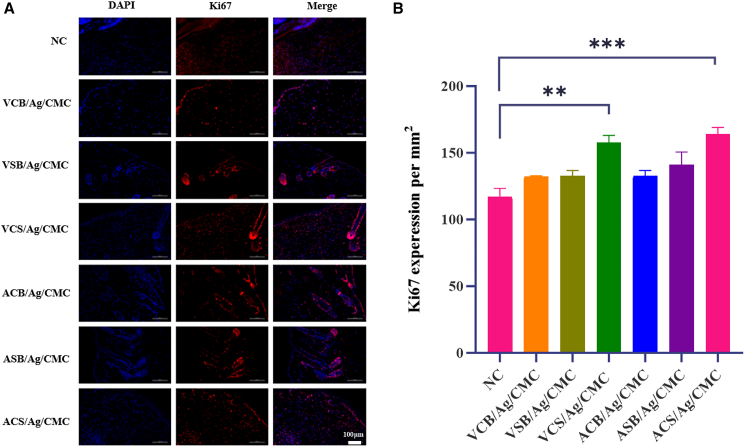
Ki67 staining to assess cell proliferation in wound sites of zwitterionic hydrogel-treated and control groups (A) Immunofluorescent staining of wound sites on day 7 post-wounding, showing Ki67-positive proliferating cells (red) and DAPI-stained nuclei (blue). (B) Quantitative analysis of the cell proliferation in the hydrogel-treated groups and the control on day 7. Data are represented as mean ± SD. Each group has 4 biological replicates (*n* = 4) ^∗^*p* < 0.05, ^∗∗^*p* < 0.01, and ^∗∗∗^*p* < 0.001.

Ki-67 is a widely recognized marker of actively dividing cells, expressed during all active phases of the cell cycle (G_1_, S, G_2_, and M) but absent in resting cells (G_0_).^54^ Evaluating Ki-67 expression provides critical insights into proliferative activity within the wound, which is a key factor for tissue regeneration, granulation tissue formation, and reepithelialization.^55^ As shown in Figure 7A, the control group exhibited the least Ki-67 expression, highlighting the limited capacity of untreated wounds to support cell division and tissue regeneration. In contrast, wounds treated with zwitterionic hydrogels (VCS/Ag/CMC and ACS/Ag/CMC) demonstrated significantly higher Ki-67 expression. Strong fluorescence signals were observed throughout the granulation tissue and wound margins, indicating a large number of proliferating cells.^56^ The quantitative analysis in Figure 7B further supports this enhanced proliferative activity. This heightened cellular activity suggests that the zwitterionic hydrogels promote robust tissue regeneration and facilitate accelerated wound healing.

**Figure 8:**
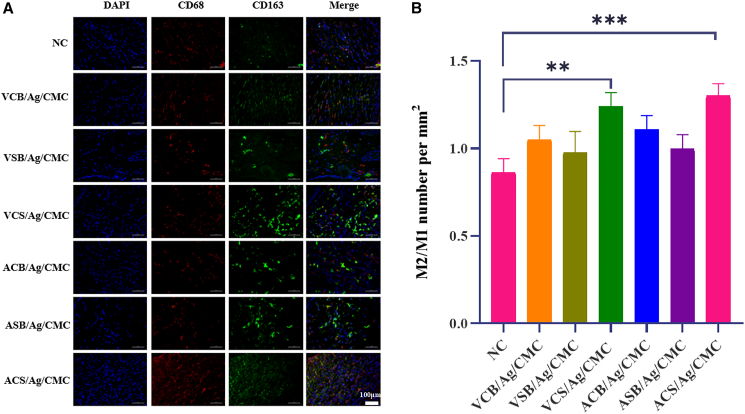
Immunofluorescent staining and quantitative evaluation of macrophage polarization and cell proliferation in wound sites of zwitterionic hydrogel-treated and control groups (A) Immunofluorescent staining of wound sites on day 7 post-wounding, showing M1 macrophages (CD68, red), M2 macrophages (CD163, green), and DAPI-stained nuclei (blue) (scale bars = 100 μm). (B) Quantitative analysis of the M2/M1 macrophage ratio in the hydrogel-treated groups and the control on day 7. Data are represented as mean ± SD. Each group has 4 biological replicates (*n* = 4). ^∗^*p* < 0.05, ^∗∗^*p* < 0.01, and ^∗∗∗^*p* < 0.001.

Immunofluorescence staining of CD68 and CD163 was conducted to assess macrophage activity during wound healing, highlighting their dual role in inflammation and tissue repair.^57^ CD68 serves as a marker for total macrophages, encompassing both pro-inflammatory M1 and anti-inflammatory M2 subtypes, reflecting overall macrophage presence in the wound environment. CD163 is a specific marker for M2 macrophages, which are crucial for resolving inflammation, promoting tissue remodeling, and stimulating angiogenesis. As shown in Figure 8A, distinct patterns of CD68 and CD163 expression were observed across the experimental groups. The quantified results (Figure 8B) compared the expression of M1 macrophages (CD68, red) and M2 macrophages (CD163, green) among the six hydrogel-treated groups and the control group. The results demonstrated that wounds treated with zwitterionic hydrogels (VCS/Ag/CMC and ACS/Ag/CMC) exhibited a significantly higher upregulation of M2 macrophages on day 7 compared to both the control group and hydrogels containing single zwitterionic functional groups.

The zwitterionic hydrogels group, in particular, showed robust CD68 and CD163 signals, underscoring their superior immunomodulatory properties. These hydrogels appear to create a microenvironment conducive to macrophage recruitment and polarization toward the M2 phenotype.^58^ Their enhanced hydration and zwitterionic functionalities likely reduce inflammatory stress, creating conditions that support tissue repair. The predominance of CD163-positive M2 macrophages in the zwitterionic hydrogel groups is especially significant, as these cells are central to resolving inflammation, promoting angiogenesis, and facilitating collagen deposition—key processes in effective wound healing.^59^

## Discussion

This study reports the successful synthesis and first-time evaluation of zwitterionic hydrogels incorporating both aliphatic and aromatic components with carboxybetaine and sulfobetaine functionalities, further enhanced by the inclusion of nanowires. The innovative design integrates the unique properties of zwitterionic hydrogels (VCS/Ag/CMC and ACS/Ag/CMC), which have been thoroughly characterized, creating a hydrogel system with superior bioactive properties. These two zwitterionic hydrogels exhibit a synergistic effect due to the presence of both positively and negatively charged groups within a single molecule, which helps maintain a sterile environment and prevent infection in wound care. Additionally, their high moisture retention capacity, facilitated by the interaction of zwitterionic polymers with water molecules through electrostatic forces and hydrogen bonding, sustains a hydrated wound environment that accelerates healing. Moreover, the incorporation of nanowires enhances the conductivity of the hydrogels, which is beneficial in applications such as tissue regeneration, where electrical signals play a crucial role in cellular behavior and growth.

Our results demonstrate that the hydrogels (VCS/Ag/CMC and ACS/Ag/CMC) exhibit remarkable antibacterial and antioxidant activities *in vitro*, highlighting their potential to mitigate infections and oxidative damage. *In vivo* evaluations showed that the hydrogels accelerate wound healing by promoting re-epithelialization, enhancing collagen deposition, and stimulating neovascularization. The hydrogels also influence macrophage polarization, promoting the M2 (anti-inflammatory) phenotype over the M1 (pro-inflammatory) phenotype. This shift is critical for reducing inflammation and enhancing tissue remodeling, as evidenced by the increased presence of M2 macrophages in the hydrogel-treated wounds. The acrylamide-based zwitterionic hydrogel (ACS/Ag/CMC) demonstrated the best performance. This groundbreaking synthesis and characterization represent a significant advancement in the field of wound care biomaterials. This study not only establishes the therapeutic potential of zwitterionic hydrogels but also lays the foundation for the further exploration of their applications in regenerative medicine.

### Limitations of the study

Although this study has been relatively perfect, there are still some limitations. First, the cell model is relatively simple, and only mouse fibroblasts (L929) were used for *in vitro* cytotoxicity experiments. However, wound healing involves a variety of cell synergies. This study lacks direct compatibility evaluation of key cells (such as keratinocytes, endothelial cells) and immune cells, which makes it difficult to comprehensively reflect the impact of hydrogels on various cells in the wound microenvironment.

The second is the lack of interpretation of the immune regulation mechanism: an important finding of the study is that hydrogels can promote macrophages to polarize toward a reparative M2 phenotype, but this is only characterized by the fluorescent staining of CD68 and CD163. The specific molecular mechanism of its regulation of polarization (such as whether it affects specific signaling pathways, such as STAT6) has not been explored in depth, making the mechanism of this interesting phenomenon slightly weak.

Finally, when designing the wound model, we only used KM male mice aged 6–8 weeks for wound modeling. Therefore, it remains to be explored whether gender will affect the research results.

## Resource availability

### Lead contact

Further information and requests for resources and reagents should be directed to and will be fulfilled by the lead contact, Dr. Udayakumar Veerabagu (udayakumar.veerabagu@emu.ee).

### Materials availability

This study did not generate new unique reagents.

### Data and code availability

• All data reported in this article will be shared by the lead contact upon request.
• This study did not report original code.
• Any additional information required to reanalyze the data reported in this article is available from the lead contact upon request.

## Acknowledgments

This work was supported by 10.13039/501100003453Natural Science Foundation of Guangdong Province (2026); Key Research Project of High Education of Guangdong Province (2024ZDZX2084). U.V. and F.Q. gratefully acknowledge the support from the ANID-FONDECYT Project No. 3200207 and No. 1200675, Chile. We thank Home for Researchers (www.home-for-researchers.com). We drew the graph by Figdraw (https://www.figdraw.com).

## Author contributions

Kit-Leong Cheong: investigation, data curation, and writing - original draft. Te Pan, Suresh Veeraperumal, Franck Quero, Gowsika Jaikumar, Timo Kikas, and Malairaj Sathuvan: investigation, data curation, and writing - review and editing. Karsoon Tan: investigation, conceptualization, data curation, and writing - review and editing. Saiyi Zhong and Udayakumar Veerabagu: conceptualization, methodology, and supervision. All authors reviewed and approved the final article.

## Declaration of interests

The authors declare no competing interests.

## STAR★Methods

### Key resources table

**Table:** 

REAGENT or RESOURCE	SOURCE	IDENTIFIER
**Antibodies**		

Anti-CD31 antibody	Servicebio	Cat.No.: GB15063；RRID:AB_3668774
Anti-Ki67 primary antibody	Servicebio	Cat.No.:GB121141；RRID:AB_3083641
Anti-CD68 antibody	Servicebio	Cat.No.:GB153150；RRID:AB_3714585
Anti-CD163 antibody	Servicebio	Cat.No.:GB15340；RRID:AB_3719564
Secondary antibodies conjugated with Alexa Fluor 488	Jackson ImmunoResearch	Cat# 715-545-150；RRID:AB_2340846
Secondary antibodies conjugated with Alexa Fluor 594	Jackson ImmunoResearch	Cat# 715-585-150；RRID:AB_2340854

**Bacterial and virus strains**		

Escherichia coli	American Type Culture Collection	ATCC 25922
Staphylococcus aureus	American Type Culture Collection	ATCC 25923

**Chemicals, peptides, and recombinant proteins**		

4-vinylpyridine	Merck	Cat# V3204
β-butyrolactone	Merck	Cat# 219126
1,3-propane sultone	Merck	Cat# P50706
N-[3-(dimethylamino)propyl]methacrylamide	Merck	Cat# 409472
Acrylic acid	Merck	Cat# 147230
1-(3-dimethylaminopropyl)-3-ethylcarbodiimide hydrochloride (EDC·HCl)	Merck	Cat# E6383
4-dimethylaminopyridine (DMAP)	Merck	Cat# 107700
Silver nanowires	Merck	Cat# 778095
Ammonium persulfate (APS)	Merck	Cat# A3678
Tetramethylethylenediamine(TMEDA)	Merck	Cat# 411019
Dulbecco’s modified eagle medium(DMEM)	Gibco	Cat# 11965092
Reagent [3-(4,5-dimethylthiazol-2-yl)-5-(3-carboxymethoxyphenyl)-2-(4-sulphophenyl)-2H-tetrazolium] (MTS)	Promega	Cat# G3580
2,2-diphenyl-1-picrylhydrazyl (DPPH)	Sigma-Aldrich	Cat# 300267
2,2-azino-bis(3-ethylbenzothiazoline-6-sulfonate) (ABTS)	Sigma-Aldrich	Cat# 194430
Penicillin-streptomycin	Gibco	15140122
Trypsin-EDTA	Gibco	25200072
Fetal bovine serum (FBS)	Gibco	A5256501

**Experimental models: Cell lines**		

Mouse fibroblast cell line (L929)	Shanghai Cell Bank of the Chinese Academy of Sciences	N/A

**Experimental models: Organisms/strains**		

Male KM mice, 6–8 weeks old	Zhuhai BesTest Bio-Tech Co., Ltd.	N/A

**Software and algorithms**		

Grapad Prism 9.3	GraphPad Software	https://www.graphpad-prism.cn/
Image J	National Institutes of Health	https://imagej.nih.gov/ij/
Image-Pro Plus 7.1	Media Cybernetics	https://my.mediacy.com/support/updates
Figdraw	Home for Researchers	https://www.figdraw.com

### Experimental model and study participant details

In this experiment, L929 cell line, *E. Coli* and *S. aureu*s were used for the cytocompatibility experiment and antibacterial performance of hydrogels, respectively. KM mice were applied to the *in vivo* experiments of hydrogels. KM mice are male mice aged 6-8 weeks, weighing 30 ± 3G. They were used after being approved by the experimental animal center of Guangdong Ocean University (gdou-lae-2022-039). All animal treatments and experiments were carried out in strict accordance with the guide for the care and use of laboratory animals of the National Research Committee, and all experimental protocols were in line with arrive guidelines. Mice were fed *ad libitum* with a standard diet and housed at 25C with a 12-h light / dark cycle and 60% humidity.

### Method details

#### Preparation of three types of aromatic silver-functionalized zwitterionic hydrogels

##### Synthesis of Acrylate-CMC (A-CMC)

Acrylic acid (0.1 mL), EDC·HCl (0.3 g), and DMAP (0.04 g) were dissolved in 2 mL of DMSO and allowed to react for 24 h. The resulting solution was then added to a 30 mL phosphate buffer solution containing CMC (1.0 g). The reaction mixture was stirred at room temperature for an additional 24 h. The product was purified through continuous dialysis (molecular weight cut-off: 7 kDa) for 24 h and subsequently lyophilized to obtain acrylate-CMC (A-CMC) is outlined in Scheme 2A, as described by Gao et al.^60^

Grafting of acrylate onto CMC was confirmed by ^1^H and ^13^C NMR (Figures S1 and S2). In the ^1^H NMR spectrum (400 MHz, D_2_O), vinyl resonances appear at δ 6.17–5.97 (m, 2H) and δ 5.62 (d, J = 9.4 Hz, 1H), consistent with acrylate incorporation. The ^13^C NMR spectrum (101 MHz, D_2_O) shows a signal at δ 152.16 ppm, assigned to the acrylate carbonyl (C=O), and peaks at δ 134.00 and 133.15 ppm corresponding to the vinyl carbons (CH and CH_2_), further corroborating successful grafting onto the CMC backbone. The degree of substitution (DS; acrylate groups per anhydroglucose unit, AGU) was determined from ^1^H integrals using DS = (A_vinyl_/3)/(A_backbone_/7), where A_vinyl_ is the summed area of the three vinyl protons (3.26) and A_backbone_ is the area of the seven non-exchangeable glucan protons (24.33). This gives DS ≈ 0.31, i.e., ∼31% of AGUs are acrylated.

##### Synthesis of 3-(4-vinylpyridin-1-ium-1-yl)butanoate (vinylpyridine carboxybetaine - VCB)

To synthesize the compound, 4-vinylpyridine (1 g) was dissolved in 5 mL of tetrahydrofuran and cooled to 0°C. Under a nitrogen atmosphere, β-butyrolactone (0.65 g) was added dropwise, and the mixture was stirred at 0–4°C for 24 h. The reaction was performed under a nitrogen atmosphere to maintain an inert environment. The crude product was washed with ether, dried under vacuum, and characterized ^1^H and ^13^C NMR spectroscopy (Figures S3 and S4), confirming the successful formation of the desired compound. The yield of the synthesized molecule was 1.22 g.

The ^1^H NMR spectrum (400 MHz, D_2_O) showed characteristic peaks at δ 8.38 (d, J = 5.9 Hz, 2H), 7.45 (d, J = 5.9 Hz, 2H), 6.69 (m, J = 17.6, 10.9 Hz, 1H), 6.06 (d, J = 17.7 Hz, 1H), 5.54 (d, J = 10.9 Hz, 1H), 3.57 (m, J = 7.1 Hz, 1H), 1.60 (d, J = 6.8 Hz, 1H), 1.47 (d, J = 6.1 Hz, 1H), and 1.10 (d, J = 7.1 Hz, 3H). ^3^C NMR (101 MHz, CDCl_3_) δ 176.30, 151.17, 147.23, 128.03, 63.08, 55.45, 41.69, confirming the presence of the desired functional groups and the compound's successful synthesis.

##### Synthesis of 3-(4-vinylpyridin-1-ium-1-yl)propane-1-sulfonate (vinylpyridine sulfobetaine -VSB)

To synthesize the compound, 1 g of vinyl pyridine was dissolved in 5 mL of dimethylformamide (DMF). Subsequently, 1.16 g of 1,3-propane sultone in 5 mL of DMF was added dropwise to the solution. The reaction mixture was stirred at room temperature for 48 h. Upon completion, the crude product was filtered and washed 3 times with ethyl acetate. The purified product was then dried at 50°C for 1 h, yielding a pale yellow powder with a mass of 1.76 g (82%).

The identity of the product was confirmed ^1^H and ^13^C NMR spectroscopy (Figures S5 and S6). The ^1^H NMR spectrum (400 MHz, D_2_O) displayed characteristic peaks at δ 8.68 (d, J = 6.7 Hz, 2H), 7.99 (d, J = 6.7 Hz, 2H), 6.90 (m, J = 17.5, 10.8 Hz, 1H), 6.40 (d, J = 17.6 Hz, 1H), 5.92 (d, J = 10.9 Hz, 1H), 4.64 (t, J = 7.4 Hz, 2H), 4.26 (t, J = 6.3 Hz, 2H), and 2.94 (m, J = 10.2, 6.9 Hz, 2H). ^13^C NMR (101 MHz, D_2_O) δ 165.10, 154.10, 144.44, 132.07, 124.84, 59.20, 47.02, 26.07, confirming the successful synthesis of the desired compound.

##### Synthesis of vinylpyridine carboxybetaine/silver nanowire/carboxymethyl cellulose (VCB/Ag/CMC)

A-CMC (1 g) was dissolved in 24 mL of water, and a solution of silver nanowires (1 mL) was then added dropwise under constant stirring. Subsequently, VCB (0.5 g) was added to the mixture and allowed to dissolve completely. Free radical polymerization was initiated by adding APS (10 mg) as the initiator and TMEDA (5 μL) as the accelerator. The resulting hydrogel samples were transferred to Petri dishes and polymerized at 70°C in an oven for 24 h. After gelation, the hydrogels were immersed in deionized water to remove unreacted materials and impurities, ensuring the purity of the final product.

##### Synthesis of vinylpyridine sulfobetaine/silver nanowire/carboxymethyl cellulose (VSB/Ag/CMC)

A-CMC (1 g) was dissolved in 24 mL of water, and a solution containing 1 mL of silver nanowires was gradually added with continuous stirring. Subsequently, VSB (0.5 g) was added to the mixture and dissolved thoroughly. Free-radical polymerization was initiated by adding APS (10 mg) as the initiator and TMEDA (5 μL) as the accelerator. The resulting hydrogel samples were transferred to a Petri dish and polymerized at 70°C in an oven for 24 h. After gelation, the hydrogels were immersed in deionized water to remove residual unreacted materials and impurities, ensuring the purity of the final product.

#### Preparation of vinylpyridine carboxybetaine/sulfobetaine/silver nanowire/carboxymethyl cellulose (VCS/Ag/CMC)

A-CMC (1 g) was dissolved in 24 mL of water, and 1 mL of silver nanowire solution was gradually added while stirring continuously. Subsequently, VCB (0.25 g) and VSB (0.25 g) were added to the mixture and dissolved thoroughly. Free-radical polymerization was initiated by adding APS (10 mg) as the initiator and TMEDA (5 μL) as the accelerator. The hydrogel samples were subsequently poured in a Petri dish and polymerized in an oven at 70°C for 24 h. After gelation, the hydrogels were immersed in deionized water to eliminate any residual unreacted materials and impurities, ensuring a pure final product. The preparation process of VCS/Ag/CMC is outlined in Scheme 2B.

#### Preparation of three types of aliphatic silver-functionalized zwitterionic hydrogels

##### Synthesis of 3-(dimethyl(3-(3-methylbut-3-enamido)propyl)ammonio)butanoate (acrylamide carboxybetaine-ACB)

N-[3-(dimethylamino)propyl]methacrylamide (1 g) was cooled to 0°C under a nitrogen atmosphere. β-Butyrolactone (0.48 mL) was added dropwise while maintaining the nitrogen atmosphere, and the reaction mixture was stirred at 4°C for 24 h. Upon completion, the crude product was filtered, washed with ether, and dried under vacuum to obtain a white solid with a yield of 1.12 g (76%).

The product was characterized using ^1^H and ^13^C NMR spectroscopy (Figures S7 and S8), which confirmed successful synthesis by revealing characteristic peaks at various chemical shifts: ^1^H NMR (400 MHz, D_2_O) δ 5.63 (d, J = 10.7 Hz, 1H), 5.39 (d, J = 10.1 Hz, 1H), 3.80 (s, 1H), 3.35–3.26 (m, 2H), 3.24 (dd, J = 15.5, 8.6 Hz, 2H), 2.96 (s, 6H), 2.58 (dt, J = 14.6, 6.3 Hz, 2H), 2.46–2.20 (m, 2H), 2.07–1.96 (m, 1H), 1.86 (s, 3H), 1.74 (dd, J = 15.1, 7.5 Hz, 1H), 1.35 (d, J = 5.7 Hz, 3H). ^13^C NMR (101 MHz, D_2_O) δ 176.69, 172.22, 139.03, 120.95, 67.09, 60.64, 43.37, 38.42, 37.15, 25.40, 17.69, 14.05.

##### Synthesis of 3-(dimethyl(3-(3-methylbut-3-enamido)propyl)ammonio)propane-1-sulfonate (acrylamide sulfobetaine-ASB)

N-[3-(dimethylamino)propyl]methacrylamide (1 g) was dissolved in 5 mL of DMF, followed by the gradual addition of 1.16 g of 1,3-propane sultone, also prepared in 5 mL of DMF. The mixture was stirred at room temperature for 2 h. Upon completion of the reaction, the crude product was filtered and washed three times with hexane, then dried at room temperature to yield a white powder weighing 1.57 g, corresponding to a 95% yield. The identity of the product was confirmed using ^1^H and ^13^C NMR spectroscopy (Figures S9 and S10), which displayed characteristic peaks at the following chemical shifts, confirming the successful synthesis of the desired compound: ^1^H NMR (400 MHz, D_2_O) δ 5.66 (s, 1H), 5.42 (s, 1H), 3.47–3.37 (m, 1H), 3.31 (dd, J = 12.0, 5.4 Hz, 4H), 3.05 (s, 6H), 2.98–2.88 (m, 4H), 2.78 (d, J = 7.6 Hz, 3H), 2.20–2.09 (m, 1H), 2.05–1.92 (m, 1H), 1.87 (s, 3H). ^13^C NMR (101 MHz, D_2_O) δ 165.02, 138.89, 121.37, 62.25, 50.84, 47.28, 37.10, 31.38, 22.33, 18.05, 17.64.

##### Synthesis of acrylamide carboxybetaine/silver nanowires/carboxymethyl cellulose (ACB/Ag/CMC)

A-CMC (1 g) was dissolved in 24 mL of water, after which 1 mL of silver nanowire solution was added drop by drop while maintaining continuous stirring. Next, 0.5 g of acrylamide zwitterion carboxylate (ACB) was added, and the mixture was stirred until fully dissolved. Once dissolution was complete, free radical polymerization was initiated by adding 10 mg of APS and 5 μL of TMEDA. The resulting hydrogel samples were poured in a Petri dish, and the polymerization was allowed to occur at 70°C for 24 h. After gelation was complete, the hydrogels were immersed in deionized water to remove any unreacted materials and impurities, ensuring the purity of the final product.

##### Synthesis of acrylamide sulfobetaine/silver nanowires/carboxymethyl cellulose (ASB/Ag/CMC)

A-CMC (1 g) was dissolved in 24 mL of water, and 1 mL of silver nanowire solution was then added drop by drop while stirring the mixture thoroughly. Next, 0.5 g of acrylamide zwitterion sulfonate (ASB) was introduced, and the mixture was stirred until the ASB completely dissolved. Once dissolved, free radical polymerization was initiated by adding 10 mg of APS and 5 μL of TMEDA. The resulting hydrogel samples were transferred to a Petri dish, and the polymerization process was allowed to proceed at 70°C for 24 h. After gelation was complete, the hydrogels were immersed in deionized water to remove any unreacted materials and impurities, ensuring the purity of the final product.

#### Preparation of acrylamide carboxybetaine/sulfobetaine/silver nanowires/carboxymethyl cellulose (ACS/Ag/CMC)

A-CMC (1 g) was dissolved in 24 mL of water, and 1 mL of silver nanowire solution was added dropwise while stirring thoroughly. Next, 0.25 g of acrylamide zwitterion carboxylate (ACB) and 0.25 g of acrylamide zwitterion sulfonate (ASB) were added to the mixture. After complete dissolution of all components, free radical polymerization was initiated by adding 10 mg of APS and 5 μL of TMEDA. The resulting hydrogel samples were carefully transferred to a Petri dish, and polymerization was allowed to proceed at 70°C for 24 h. Once gelation was complete, the hydrogels were immersed in deionized water to remove any residual unreacted materials and impurities, ensuring the purity of the final product. The preparation process of ACS/Ag/CMC is outlined in Scheme 2C.

#### Characterization

The phase purity of the catalyst was assessed using wide-angle X-ray diffraction (XRD) on a Bruker D8 diffractometer (AXS, Germany) with Cu Kα radiation. The hydrogel samples were fully dried to remove any residual water, ground into a fine powder, and placed into an XRD sample holder. The scans were performed over a 2θ range of 10° to 80° to obtain the diffraction pattern. For morphological and elemental analysis, field emission scanning electron microscopy (FESEM) and energy-dispersive X-ray spectroscopy (EDX) mapping were conducted using a Gemini SEM 300. The dehydrated samples were dried, cut into smaller pieces to fit the SEM holder, and coated with a thin conductive layer of carbon using a sputter coater. The samples were then placed in the SEM chamber for both morphological and EDX analysis. The functional groups within the hydrogels were characterized using Fourier transform infrared spectroscopy (ATR-FTIR) on a Nicolet 6700 instrument (USA). A small piece of the film was placed directly onto the ATR crystal, and the sample was subjected to FTIR analysis. The silver content in the hydrogels was quantified using inductively coupled plasma–optical emission spectrometry (ICP-OES) with a PerkinElmer Optima 5300DV instrument, and the results were expressed as weight percentage. Nuclear magnetic resonance (NMR) spectra were obtained using a Bruker AVANCE III HD-400 MHz spectrometer with deuterated solvents. The samples were dissolved in D2O, placed into an NMR tube, and loaded into the spectrometer for analysis.

#### Mechanical strength

The mechanical properties of the hydrogels were measured using a universal testing machine equipped with a low-capacity load cell designed for soft biomaterials. Hydrogel samples were prepared in standardized dumbbell-shaped molds, thoroughly rinsed with distilled water to remove unreacted species, and subsequently equilibrated in 1×PBS at 37°C for at least 12 h before testing. Tensile experiments were carried out at a constant crosshead speed of 300 mm/min, and the average values of tensile strength and elongation at break were calculated from three independent replicates.^61^

#### Swelling study

The swelling ratio of the hydrogels were measured in both distilled water and phosphate buffer solutions. The hydrogel samples were prepared in identical molds and thoroughly washed in distilled water to remove unreacted species. Washed samples were frozen (−80°C, 2 h), and lyophilized, and the constant mass of all dried samples were immersed in parallel in distilled water and phosphate buffer at 37°C for 48 h to reach maximum swelling equilibrium. The swelling ratio was calculated according to the following equation:

$$\text{Swelling}\;\text{ratio}\;\left(\%\right)=\left(W_{2}-W_{1}\right)/W_{1}\times 100$$

W_1_ is initial dry weight of the hydrogel, and W_2_ is the weight of the hydrogel after swelling.^62^

#### Degradation rate

The degradation behavior of the hydrogels was evaluated in phosphate buffer solution at 37°C over 28 days. Dried hydrogel samples of constant initial weight were immersed in PBS and incubated in a shaker at 70 rpm/min. Separate sets of samples were prepared for each time point (7, 14, 21, and 28 days).^63^ At the designated intervals, samples were removed, gently washed with distilled water, lyophilized, and weighed to determine the remaining mass. The degradation rate was calculated using the following equation:

$$\text{Weight}\;\text{remaining}\;\text{ratio}\;\left(\%\right)=W_{2}/W_{1}\times 100$$

W_1_ is initial dry weight of the hydrogel, and W_2_ is the dry weight after degradation at each time point.

#### Silver-ion release behavior

The release of silver ions from the hydrogels was evaluated by incubating square samples (1 × 1 cm^2^) in 10 mL of phosphate buffer solution at 37°C. At predetermined time intervals, the release medium was collected and replaced with an equal volume of fresh PBS to maintain sink conditions.^64^ The collected aliquots were digested with 5% nitric acid, and the silver concentration was quantified using ICP-OES.

#### Determination of cell viability

Mouse fibroblasts L929 were used for cytocompatibility evaluation consistent with our in-vitro design (24 h MTS assay). Cells were cultured in Dulbecco's Modified Eagle Medium (DMEM, high glucose, 4.5 g L^-1^) supplemented with 10% (v/v) fetal bovine serum (FBS) and 1% (v/v) penicillin-streptomycin (100 U mL^-1^/100 μg mL^-1^). Cultures were maintained at 37°C in a humidified atmosphere of 5% CO_2,_. Cells were passaged at ∼80% confluence using 0.25% trypsin-EDTA for 2-3min, neutralized with complete medium, centrifuged (300 ×g, 5 min), and released at the desired density.^65^

Approximately 1 × 10^4^ L929 fibroblast cells were seeded in 96-well plates and incubated for 24 h to reach ∼70% confluency.^66^ To evaluate cytocompatibility under maximum-exposure conditions, the hydrogels were solubilized at different concentrations (0–2 wt%) in complete DMEM medium, thereby representing the highest possible levels of soluble components and degradation products. After washing the cells with ice-cold PBS, the hydrogel solutions were added and incubated for 24 h. Cell viability was assessed using the MTS assay according to the manufacturer’s protocol. Following treatment, 20 μL of MTS reagent was added to each well and incubated for 1 h at 37°C in a humidified 5% CO_2_ atmosphere. Absorbance was recorded at 490 nm using a microplate reader, and results were expressed as percentage viability relative to untreated controls.

#### Antibacterial performance evaluation

Using a UV–vis spectrophotometer (Evolution 220 UV–vis, Thermo Scientific, USA) the antibacterial efficacy of the hydrogel against Escherichia coli (ATCC 25922) and Staphylococcus aureus (ATCC 25923) was evaluated by monitoring bacterial growth by optical density measurement at 600 nm. The two bacterial strains were cultured in LB broth at 37° C under aerobic conditions until reaching the exponential growth period, and the bacterial concentration was normalized to about 1 × 10^6^ CFU/ml.^67^ Sterilized hydrogel discs (10 mm in diameter and 2 mm in thickness) were prepared by UV irradiation for 30 min. For the assay, the experimental group consisted of bacterial suspensions incubated with different hydrogel discs. The control group (negative control) consisted of bacterial suspensions without any hydrogel treatment, representing normal bacterial growth. Specifically, hydrogel discs were immersed in 5 ml of sterile LB broth containing bacterial suspension. The control group contained equal volumes of broth and bacteria without hydrogel discs. All samples were incubated at 37° C for 24 h with gentle shaking at 100 rpm. After incubation, the OD600 value of the suspension was measured using a microplate reader. The antibacterial rate was calculated by comparing the OD value of the experimental group with that of the negative control.

#### Antioxidant assay

The antioxidant activity of the silver-functionalized zwitterionic hydrogels was assessed using the DPPH and ABTS *in vitro* assays.

For the DPPH assay, a fresh 0.1 mmol/L DPPH solution in ethanol was prepared and stored in the dark. Hydrogel samples (10 mg) were immersed in 3 mL of the DPPH solution and kept in the dark at room temperature for 30 min. A negative control, containing only the DPPH solution without any hydrogel sample, was prepared in parallel. The absorbance at 517 nm was measured for both the samples and the control. The DPPH radical scavenging activity was calculated using the following formula:^68^

For the ABTS assay, the radical cation was generated by reacting a 7.4 mmol/L ABTS stock solution with 2.6 mmol/L potassium persulfate in the dark for 24 h at room temperature. Hydrogel samples were then mixed with 3 mL of the resulting ABTS solution and incubated in the dark for 20 min. Similarly, a negative control containing only the ABTS solution was used. The absorbance at 734 nm was measured, and the ABTS radical scavenging activity was calculated using the same formula as for the DPPH as.^69^

#### Assessment of wound healing process

The wound healing activity *in vivo* was assessed using modified protocols based on established methodology with minor modifications.^70^ Male KM mice (6–8 weeks old, n=56,weight = 30 ± 3 g) were utilized after approval was granted by the Experimental Animal Centre, Guangdong Ocean University (GDOU-LAE-2022-039). All the animal treatments and experiments were performed in strict accordance with the Guidelines for the Care and Use of Laboratory Animals of the National Research Council, and all the experimental protocols adhered to the ARRIVE guidelines. The mice were fed *ad libitum* with a standard diet and housed at 25°C with a 12-hour light/dark cycle and 60% humidity. Circular wounds (0.5 cm in diameter) were created on the shaved dorsal area of anesthetized mice. Following the procedure, the animals were randomly divided into seven experimental groups (n = 8 per group): a control group without treatment (NC), three groups treated with aromatic silver-functionalized zwitterionic hydrogels (VCB/Ag/CMC, VSB/Ag/CMC, VCS/Ag/CMC), and three groups treated with aliphatic silver-functionalized zwitterionic hydrogels (ACB/Ag/CMC, ASB/Ag/CMC, and ACS/Ag/CMC). Hydrogel dressings, pre-cut into 7 mm diameter discs, were immediately applied to the wound sites. To prevent contamination, the dressings were secured with a Tegaderm transparent film (3M Health Care, Germany) and wrapped with a thin, self-adhesive bandage to maintain stability.

Digital images of the wounds were taken to document initial wound size and track healing progress. Measurements of wound areas were conducted at specific intervals (days 0, 4, 7, 10, 14, and 17), and the percentage of wound closure was calculated as the reduction in wound area relative to the baseline. The wound margins were delineated using Image-Pro Plus 7.1 software for precise measurements. On days 7 and 14 post-surgery, the mice were anesthetized with 4% chloral hydrate, euthanized via cervical dislocation, and skin tissue, including the wound area, was collected for subsequent analyses.

#### Histologic analysis

The experimental mice were sacrificed on days 7 and 14, after which the injured tissues were collected, fixed in a 10% formalin solution, and embedded in paraffin. Tissue sections with a thickness of 4 μm were prepared for subsequent analysis. Wound healing progression was assessed through hematoxylin and eosin (H&E) staining and Masson's trichrome staining, which were used to evaluate collagen deposition. The stained tissue samples were examined under a light microscope (Nikon Eclipse E100, Nikon, Japan) at 50× magnification, and images were captured for further analysis.

#### Immunofluorescence staining

Tissue sections (5 μm thick) were deparaffinized with xylene, rehydrated through a graded ethanol series, and rinsed in PBS. Antigen retrieval was performed by immersing the sections in sodium citrate buffer (10 mmol/L, pH 6.0) at 95°C for 15 minutes, then allowing them to cool to room temperature. The sections were then washed with PBS and blocked with 5% bovine serum albumin in PBS containing 0.1% Tween-20 for 1 h at room temperature to minimize nonspecific binding. Subsequently, the sections were incubated overnight at 4°C with primary antibodies targeting CD31 (1:200), Ki67 (1:200), CD68 (1:200), and CD163 (1:200). Following primary antibody incubation, the sections were rinsed three times with PBS and treated with fluorescently labeled secondary antibodies (Alexa Fluor 488 and Alexa Fluor 594, 1:400) for 1 h at room temperature in a dark environment. Nuclei were counterstained using 4',6-diamidino-2-phenylindole (DAPI), and the sections were mounted with an antifade medium. Negative controls were prepared by omitting the primary antibody to confirm staining specificity.

Images were captured using a laser scanning confocal microscope (Nikon Eclipse C1, Japan), employing lasers optimized for DAPI (blue), Alexa Fluor 488 (green), and Alexa Fluor 594 (red). CD31 expression, indicative of microvascular density, was quantified as the proportion of CD31-positive staining relative to the total tissue area. Ki67 expression, used as a marker of cell proliferation, was evaluated by calculating the ratio of Ki67-positive nuclei to total nuclei. Quantitative analysis of CD68 and CD163 staining was performed with ImageJ software. CD68 marked macrophage infiltration, while CD163 identified M2-polarized macrophages. The percentage of positively stained cells or fluorescence intensity relative to the total tissue area was determined based on five randomly selected fields per section.

### Quantification and statistical analysis

All experiments were performed in triplicates, and experimental data are shown as mean ±SD. The biological replicates (n) of each experiment are indicated in the corresponding figure legends. Grapad prism 9.3 software was used for statistical analysis of the experimental data, and one-way ANOVA followed by the Tukey test was used for statistical analysis. P-value < 0.05 were considered statistically significant. Quantitative analysis of histological and immunofluorescence images was performed using Image J software. For collagen deposition in Masson trichrome-stained sections, the blue-stained collagen area was quantified by applying a consistent color threshold to distinguish blue (collagen) in all images. The percentage of collagen area was calculated as (blue pixel area / total tissue area) × 100%. For immunofluorescence analysis (such as CD31, Ki-67), the wound area was randomly selected as the region of interest. The fluorescence intensity threshold was set to distinguish positive staining from background, and the threshold of all samples within the same experiment was consistent. Subsequently, the percentage of positive area or the number of positive cells were automatically calculated by the software.
