# Supplementary material for: Multifunctional carboxymethyl cellulose-based hydrogels with zwitterionic and silver nanowire components for wound management
Source: iScience. 2025 Dec 4;29(1):114336. doi: 10.1016/j.isci.2025.114336 (PMC12775929; doi:10.1016/j.isci.2025.114336)
Supplement: Document S1. Figures S1–S11 [file mmc1.pdf]

iScience, Volume *29*

Supplemental information

Multifunctional carboxymethyl cellulose-based hydrogels with zwitterionic and silver nanowire components for wound management

Kit-Leong Cheong, Te Pan, Suresh Veeraperumal, Franck Quero, Gowsika Jaikumar, Timo Kikas, Malairaj Sathuvan, Karsoon Tan, Saiyi Zhong, and Udayakumar Veerabagu

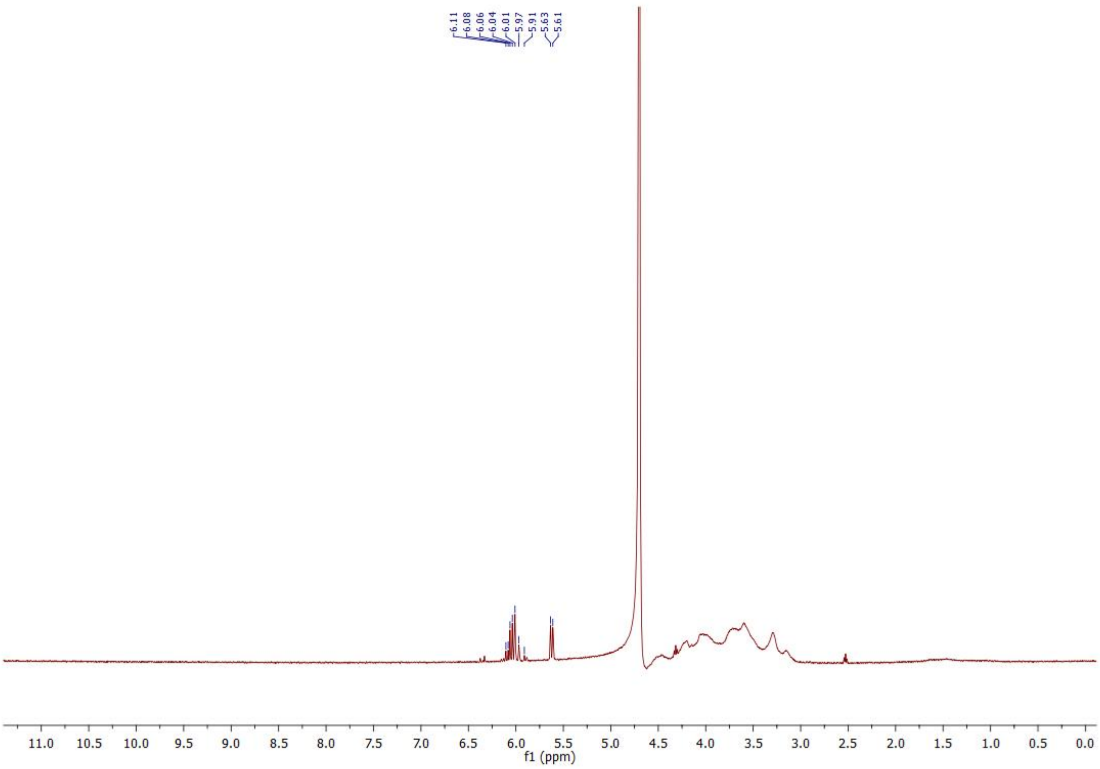

Figure S1. ^{1}H NMR spectroscopy of acrylate-CMC

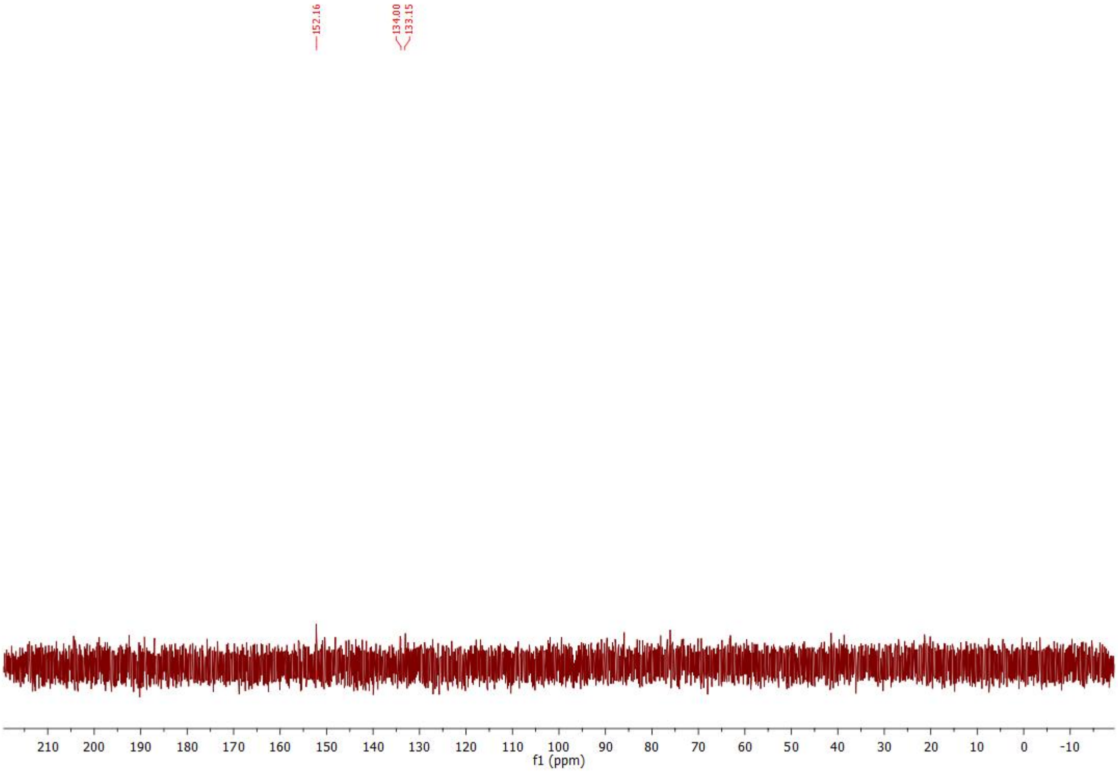

Figure S2. ^{13}C NMR spectroscopy of acrylate-CMC

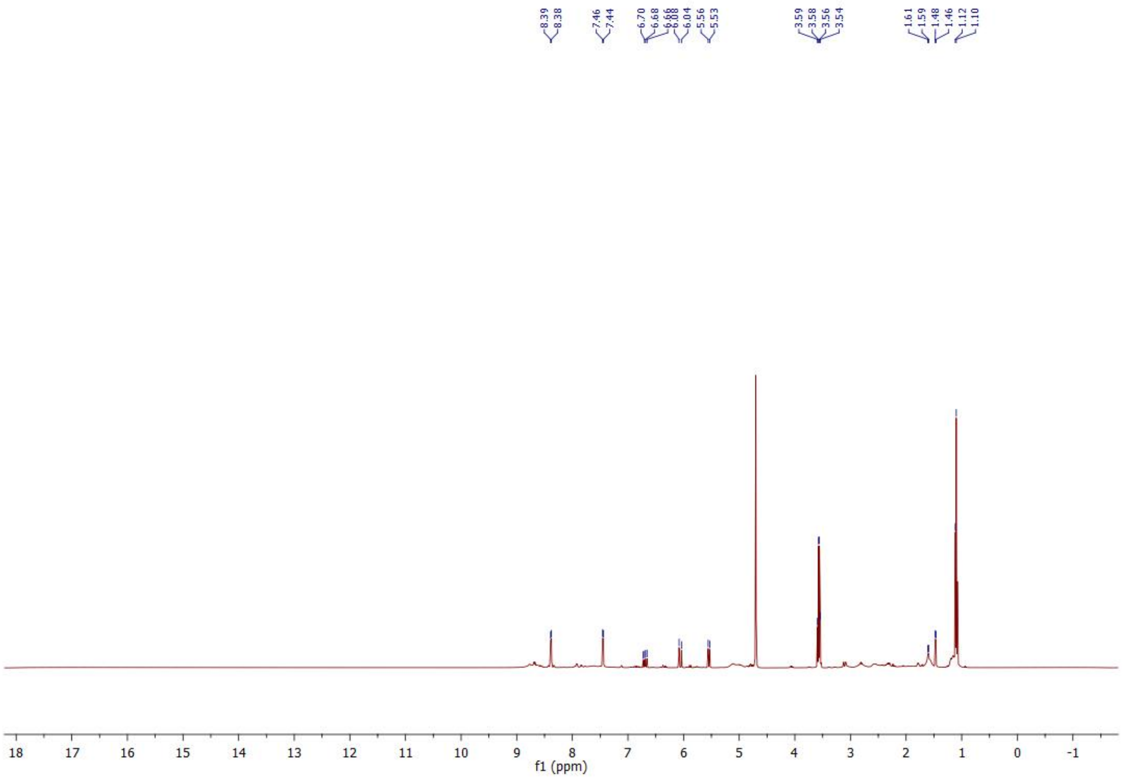

Figure S3. ^{1}H NMR spectroscopy of vinylpyridine carboxybetaine – VCB

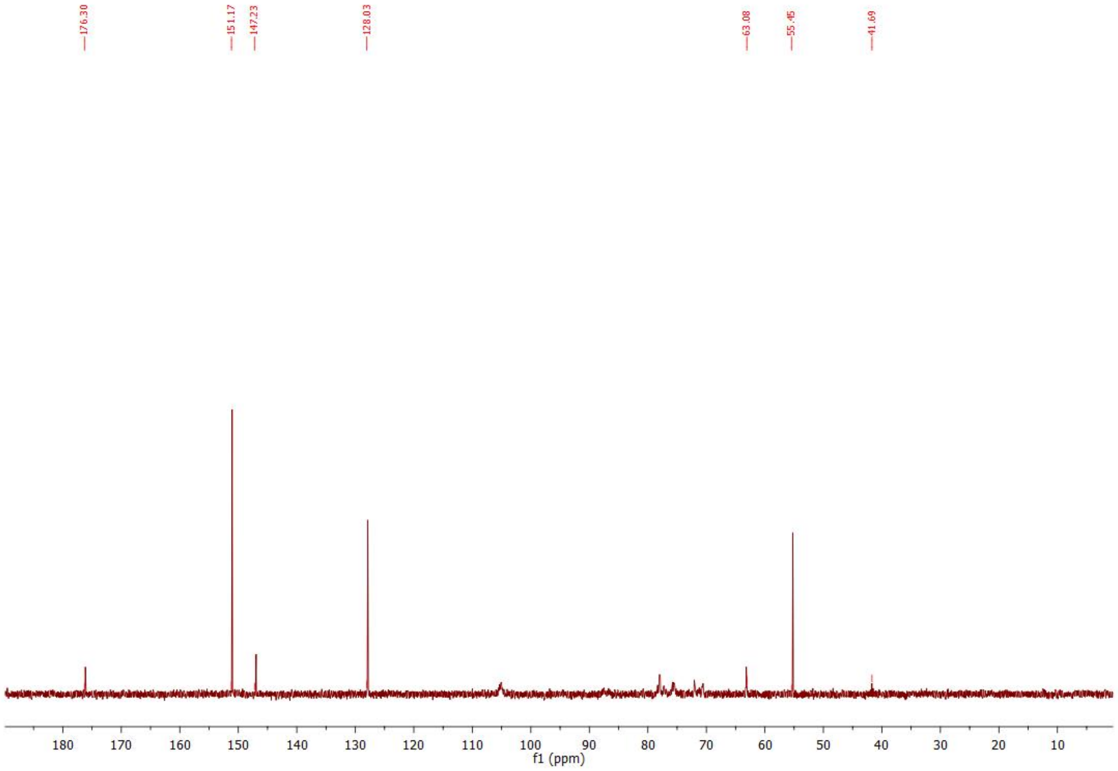

Figure S4. ^{13}C NMR spectroscopy of vinylpyridine carboxybetaine – VCB

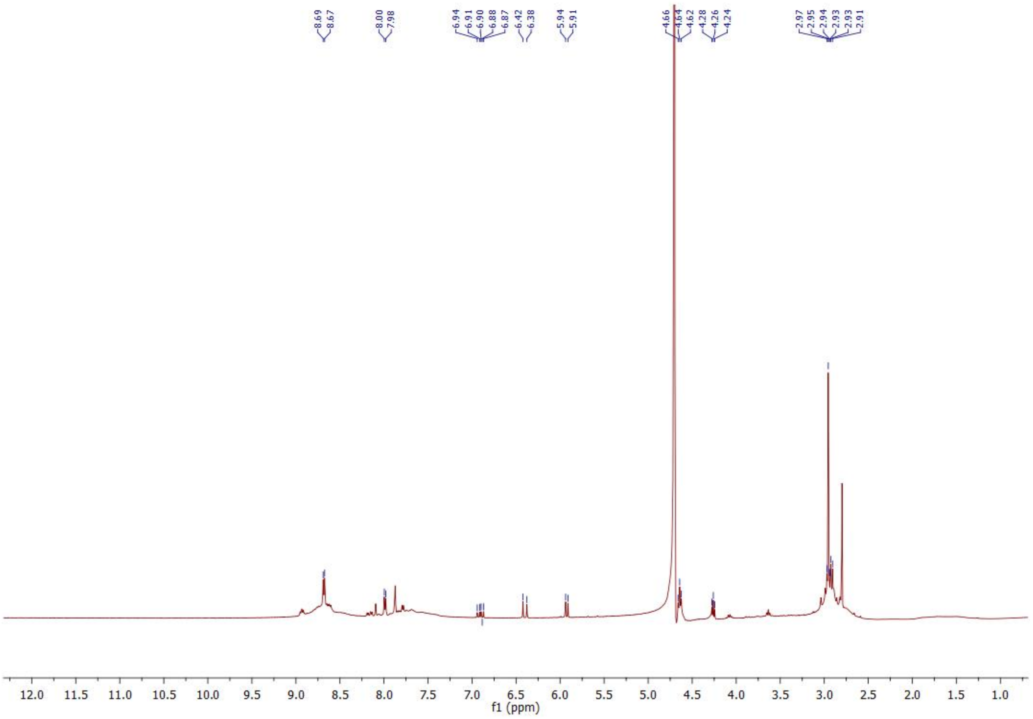

Figure S5. ^{1}H NMR spectroscopy of vinylpyridine sulfobetaine -VSB

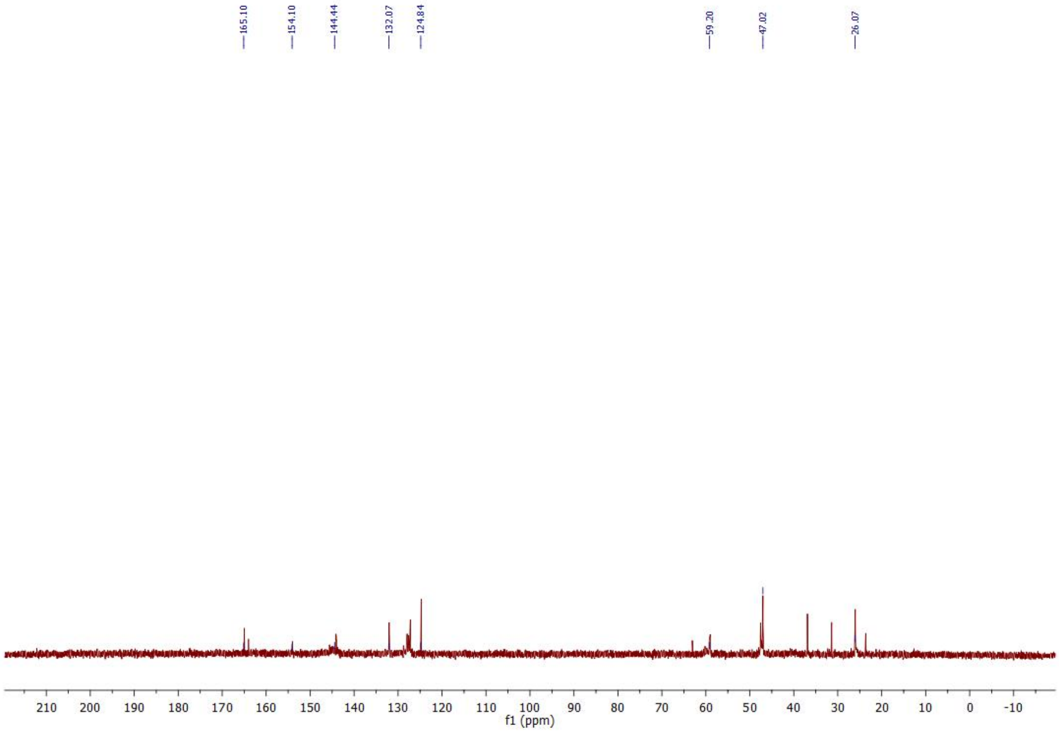

Figure S6. ^{13}C NMR spectroscopy of vinylpyridine sulfobetaine -VSB

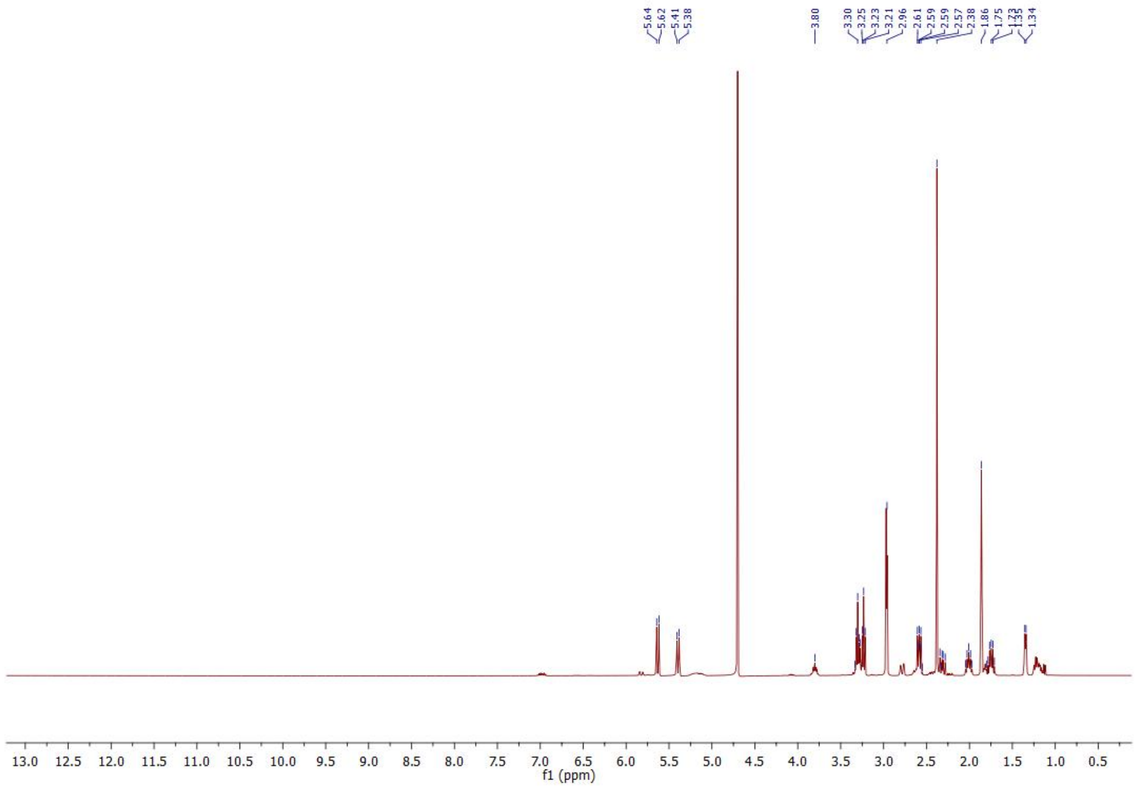

Figure S7. ^{1}H NMR spectroscopy of acrylamide carboxybetaine-ACB

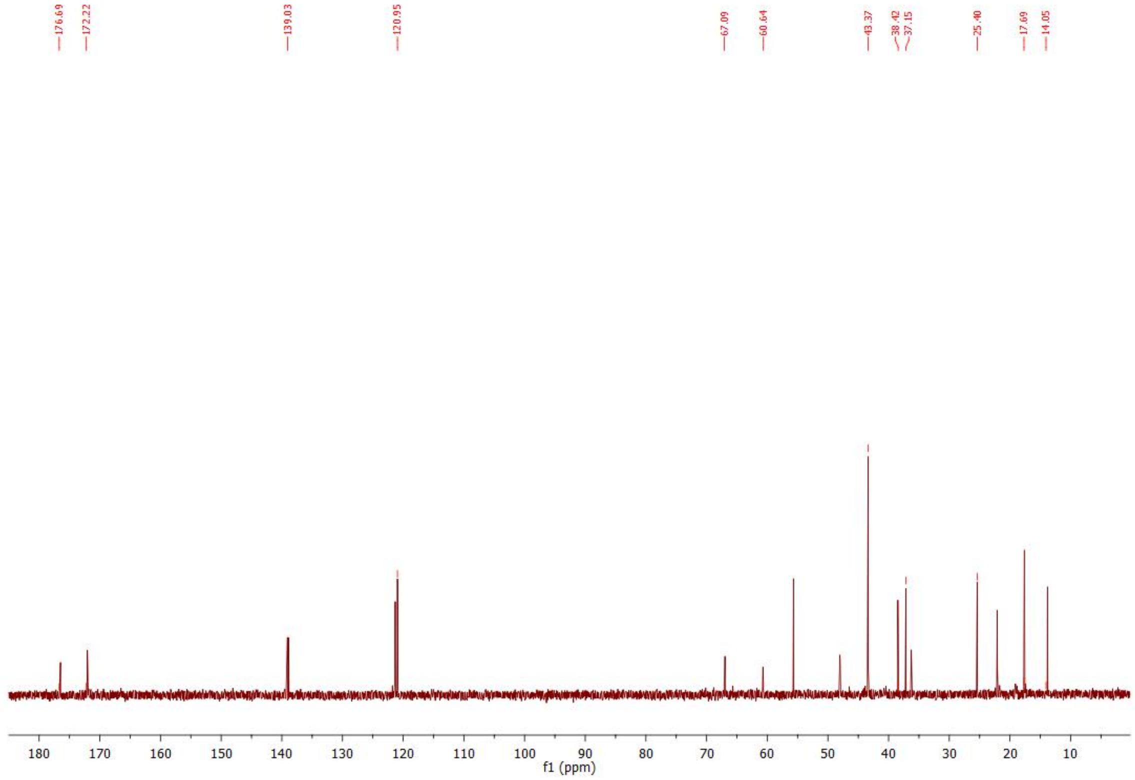

Figure S8. ^{13}C NMR spectroscopy of acrylamide carboxybetaine-ACB

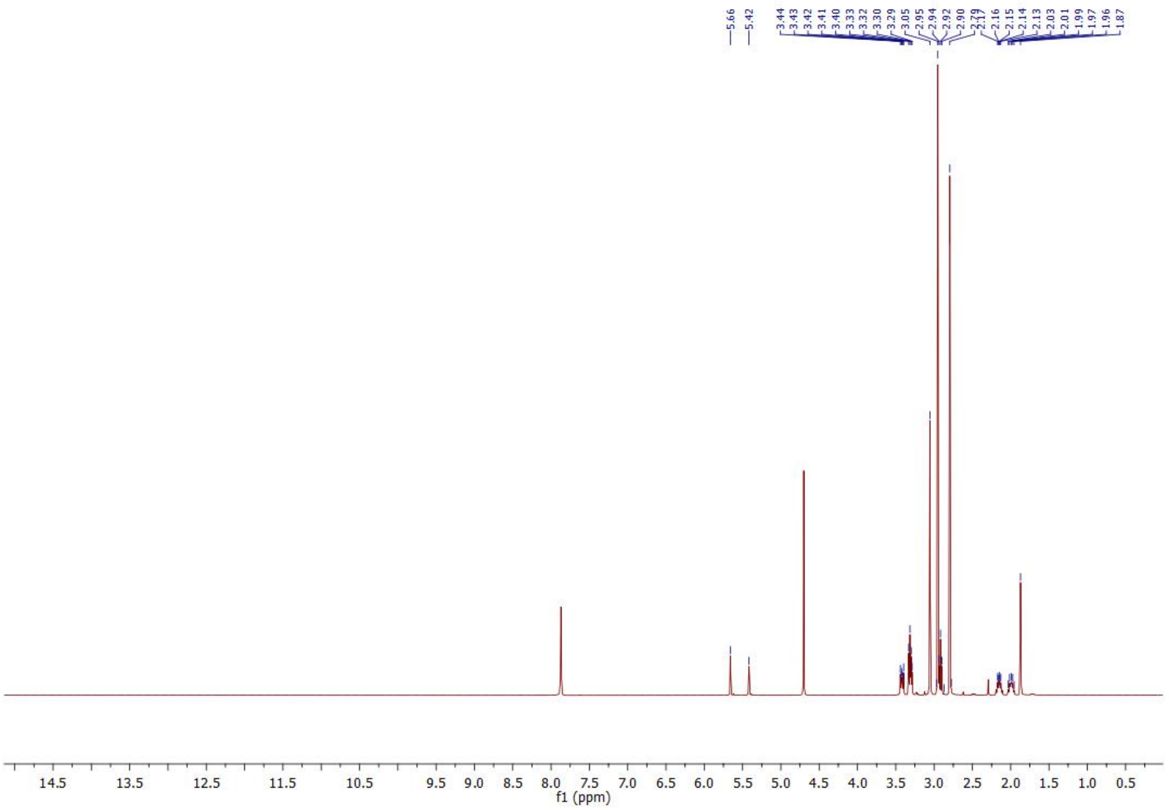

Figure S9. ^{1}H NMR spectroscopy of acrylamide sulfobetaine -ASB

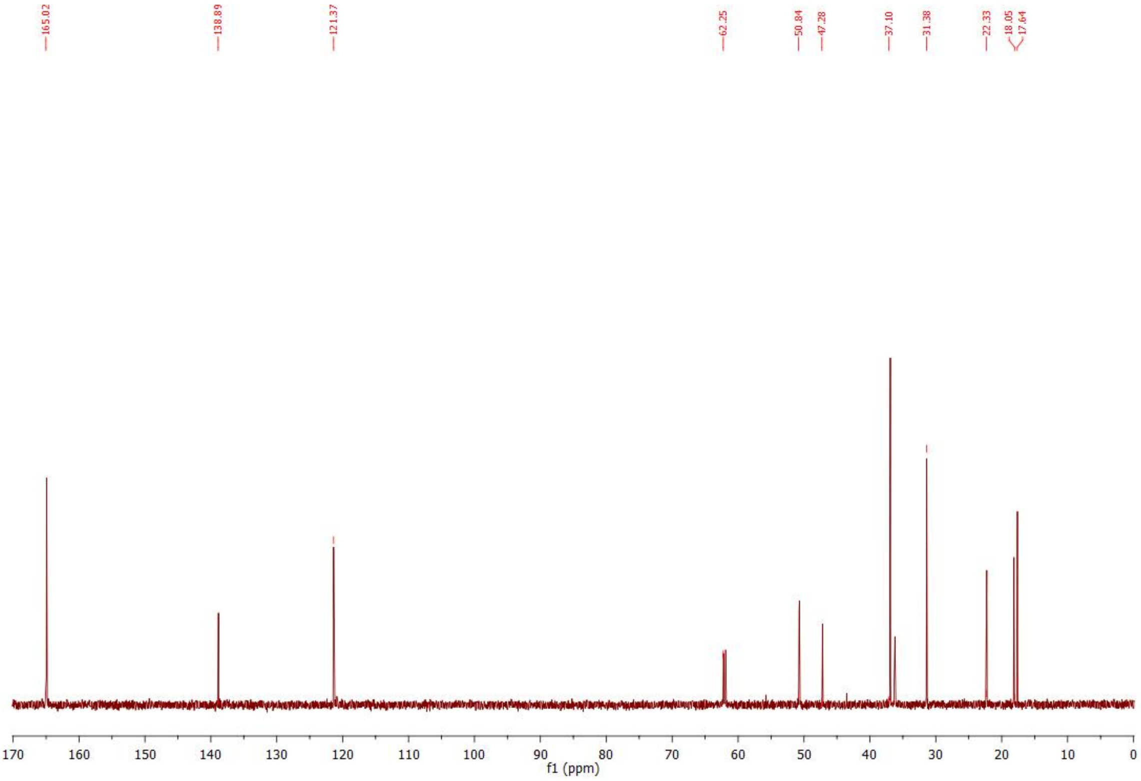

Figure S10. ^{13}C NMR spectroscopy of acrylamide sulfobetaine -ASB

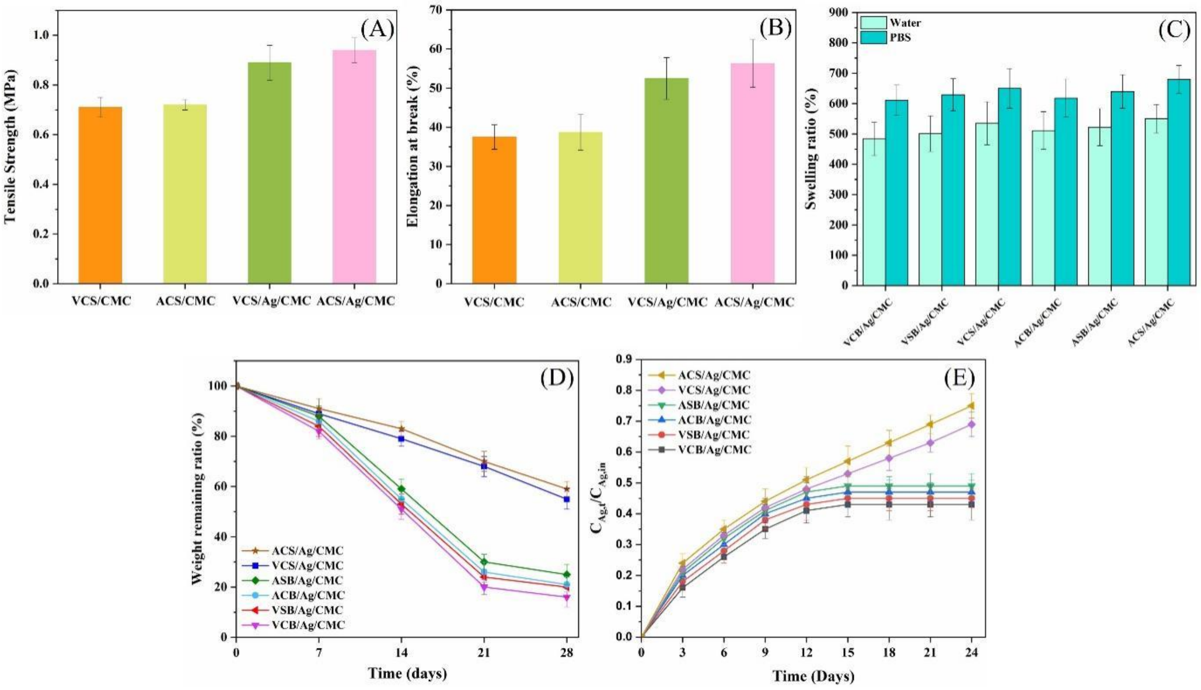

Figure S11. Physicochemical properties of hydrogels. (A) Tensile strength and (B) elongation at break of the hydrogels. (C) Swelling ratios in water and PBS up to 48 h. (D) Degradation profiles in PBS over 28 days. (E) Silver-ion release from hydrogels in PBS at 37 °C for 24 days. Data are represented as mean +/- SD, n = 6. $^{*}p < 0.05$ and $^{**}p < 0.01$.
